# Mannans as Multifunctional Biopolymers: Structure, Properties, and Applications in Health and Industry

**DOI:** 10.3390/polym17243297

**Published:** 2025-12-12

**Authors:** Isaac Karimi, Layth Jasim Mohammed, Ahmed Makki Amshawee, Nahlah Fatehi Makki, Kosar Nazari, Helgi B. Schiöth

**Affiliations:** 1 Research Group of Bioengineering and Biotechnology, Laboratory for Computational Physiology, Department of Biology, Faculty of Science, Razi University, Kermanshah 67149-67346, Iran; kosar.nazari@razi.stu.ac.ir; 2 Department of Microbiology, College of Medicine, Babylon University, Hilla City 51002, Iraq; med996.layth.jasim@uobabylon.edu.iq; 3 Department of Radiology, College of Health and Medical Technology, University of Hilla, Hilla City 51002, Iraq; ahmed_meki@hilla-unc.edu.iq; 4 Department of Chemistry, College of Science, Kufa University, Najaf 54001, Iraq; nahla.almutawalli@uokufa.edu.iq; 5 Department of Surgical Sciences, Functional Pharmacology and Neuroscience, Uppsala University, 751 24 Uppsala, Sweden

**Keywords:** mannan, biopolymer, prebiotics, nutraceuticals, drug delivery, nanocomposites

## Abstract

Mannans are structurally composed of β-(1 → 4)-linked mannose units, which are widely distributed in plant cell walls, yeast, and bacterial exopolysaccharides. Mannans have emerged as multipurpose biopolymers with significant industrial and biomedical potential. Celebrated mannans include guar gum, locust bean gum, konjac glucomannan, yeast mannans, and softwood glucomannans. This comprehensive review highlights the sources, structural diversity, extraction methods, physicochemical properties, safety, and functional characteristics. The major bioactivities of mannans, including immunomodulatory, antioxidative, and prebiotic effects, reflect their relevance in biopharmaceutical applications. Moreover, mannans serve as valuable raw materials for developing biodegradable films, hydrogels, and nanocomposites applied in sustainable materials and drug delivery systems. Despite promising applications, challenges related to their large-scale production, standardization, and functional optimization remain to be investigated. Future perspectives focus on integrating advanced biotechnological approaches and chemical modifications to enhance the functional versatility of mannans. Overall, mannans represent a sustainable, multifunctional biopolymer with expanding applications across food, pharmaceutical, and biomedical industries.

## 1. Introduction

Biopolymers are macromolecules synthesized by living organisms, and include nucleic acids, proteins, and polysaccharides. Owing to their biodegradability, renewable origin, and functional versatility, biopolymers have attracted increasing attention as sustainable alternatives to petroleum-derived polymers in various industries. Among polysaccharide biopolymers, mannans, polymers of mannose residues, stand out for their origins, structural diversity, widespread occurrence, and wide range of applications [1]. Mannans are a major component of plant hemicellulose, second only to cellulose in abundance, and are extensively distributed in softwoods, legume seeds, and microbial cell walls [2]. Structurally, they occur as linear mannans, glucomannans (mannose–glucose copolymers), galactomannans (mannose polymers with galactose branches), and acetylated mannans, reflecting considerable biochemical heterogeneity [3]. This variability alters their diverse physicochemical properties, such as viscosity, gelling, and film formation, making them useful in food technology, feed formulation, and pharmaceuticals [4]. Therefore, mannans have emerged as particularly relevant due to their widespread biological roles, structural adaptability, and expanding technological potential.

In nature, mannans perform essential biological functions across diverse taxa. In plants, mannans contribute to cell wall architecture, stress responses, and seed germination [5]. In fungi and yeasts, mannans are integral to the cell wall, contributing to immune recognition and host–pathogen interactions [6]. For example, cell wall molecules of *Candida albicans* mediate interactions with the environment and host immune cells. Recognition occurs through pathogen recognition receptors (PRRs) on neutrophils and macrophages, which bind to mural components. β-glucan and mannan trigger proinflammatory responses, whereas chitin promotes anti-inflammatory effects. Notably, *C. albicans* can modulate the exposure of these pathogen-associated molecular patterns (PAMPs) in response to environmental cues, thereby shaping host immunity (for a review, see [7]). Recently, mannans have been recognized as promising biomedical biopolymers.

Building on these intrinsic properties, mannans are now gaining attention as renewable biomaterials with significant potential for applications. Their biodegradability, biocompatibility, and low immunogenicity make them attractive for advanced applications such as drug delivery, vaccine adjuvants, and tissue engineering scaffolds [8,9]. Moreover, enzymatic degradation of mannans by mannanases produces mannan oligosaccharides (MOS), which have prebiotic effects and are widely applied in functional foods and animal nutrition [4]. Therefore, beyond their industrial relevance, mannans serve crucial biological functions and offer significant biomedical potential, with applications ranging from immunomodulation and drug delivery to prebiotic nutrition and tissue engineering.

Despite expanding applications, the field remains fragmented, with structural classifications, source diversity, physicochemical properties, and enzymatic processing often examined in isolation. This review aims to integrate these dimensions into a unified framework, providing an updated synthesis of mannan structure–property relationships, natural occurrence, biological function, enzymatic degradation pathways, and emerging technological uses. In contrast to existing reviews, we emphasize (i) cross-linking biological roles with material functionalities, (ii) recent advances in mannan-based biomedical technologies, and (iii) knowledge gaps that limit the full deployment of mannans as sustainable, high-value biopolymers. In this regard, a randomized survey of the English-language literature was performed across multiple databases, including Embase, Web of Science, PubMed, and Scopus, to find publications related to mannans. This review synthesizes insights into its chemical properties and highlights its diverse applications in various fields.

## 2. Chemical Structure and Types of Mannans

Mannans are a class of hemicellulosic polysaccharides composed mainly of β-D-mannose residues, though they frequently contain other monosaccharides such as glucose and galactose (for a review, see [10]). The mannose backbone is typically linked through β-1,4-glycosidic bonds, with occasional α-1,6- or β-1,6-linked branches, depending on biological source [3]. This structural heterogeneity created at least four major types: linear mannans, glucomannans, galactomannans, and galactoglucomannans (GGM) ([4]; Table 1). In this context, linear mannans or homomannans consist of homopolymeric chains of β-1,4-linked mannose residues and are relatively rare in nature. They occur mainly in ivory nuts (vegetable ivory) and some microbial cell walls [3]. Glucomannans are copolymers of mannose and glucose residues arranged in a β-1,4-linked backbone (e.g., [11]). They are widely distributed in softwood hemicellulose, making them a significant structural component of gymnosperm secondary cell walls [12]. Galactomannans contain a linear β-1,4-mannan backbone with α-1,6-linked galactose side chains. These are commonly found in the endosperm of legume seeds, such as guar gum (*Cyamopsis tetragonoloba*) and locust bean gum (LBG; *Ceratonia siliqua*) [13,14], and pumpkin [15]. The degree of galactose substitution influences solubility, viscosity, and gelling properties, which underpins their wide application in food, pharmaceuticals, and cosmetics [9]. GGM, the most complex subtype, contains mannose, glucose, and galactose residues, often with additional *O*-acetyl substitutions (for a review, see [16]). They are predominant in softwoods, where they contribute up to 20% of the dry weight of secondary cell walls [2]. In addition to terrestrial sources, miscellaneous mannans are present in marine organisms, where sulfated mannans occur in red and green algae (for a review, see [17]). Using techniques like small-angle neutron scattering, dynamic and static light scattering, circular dichroism, and cryo-transmission electron microscopy, the mannan was found to contain rigid-rod regions with a 14-helix conformation and strong water interactions. These structural features underpin its ice recrystallization inhibition activity, highlighting the relationship between mannan structure and function [18]. Thus, mannans represent a structurally diverse group of polysaccharides whose monosaccharide composition, glycosidic linkage pattern, branching, and substitution profile (e.g., acetylation, sulfation) are strongly determined by their biological source. This section outlines the architectural basis for mannan diversity, which underpins the physicochemical behavior discussed in Section 3 and the resulting application potential described in Section 4.

## 3. Physicochemical Properties of Mannans

The physicochemical properties of mannans are governed by their monosaccharide composition, glycosidic linkage patterns, degree of branching, and the presence of substituents such as acetyl or sulfate groups (Table 1). These structural determinants dictate their water solubility, hydration capacity, viscosity, gelling ability, and biodegradability [3,12]. For example, the ratio of mannose to other monosaccharides influences chain flexibility and solubility, while α-1,6-linked side chains or acetyl groups can disrupt intermolecular packing, altering swelling behavior and rheology. Sulfation introduces charge, which affects electrostatic interactions, hydration, and thermal stability (*vide infra*). Collectively, these structure-dependent physicochemical properties determine how mannans behave in aqueous environments, how they interact with other biomolecules, and how they perform in both biological systems and industrial formulations. Additionally, the physicochemical properties of mannans as dietary fiber in the gut, such as hydration, adsorption, and rheology, significantly affect digestion, satiety, and glucose and lipid metabolism. Due to its diverse properties, konjac glucomannan (KGM) serves as a valuable model for investigating the relationships between fiber structure, functionality, and metabolic regulation. A review discussed the role of KGM in nutrient digestion from a colloidal nutrition perspective, methods to modify its properties, the relationship between structure and metabolic effects, and its applications in fat-reducing foods, providing a theoretical basis for dietary fiber colloid nutrition research [19].

### 3.1. Solubility

Mannan solubility is strongly influenced by patterns of structural substitutions and environmental factors. Linear mannans with a homopolymeric β-1,4-mannose backbone, such as those from ivory nut, are typically insoluble in water due to extensive inter-chain hydrogen bonding [3]. In contrast, galactomannans, such as those from guar and LBG, exhibit improved solubility because α-1,6-linked galactose side chains disrupt aggregation and enhance hydration [9]. The galactose:mannose (G:M) ratio is a critical determinant of solubility and rheological behavior, with higher galactose substitution favoring dissolution [9,20,21]. Additional structural modifications, including acetylation, sulfation, or phosphorylation, further modulate solubility by introducing steric hindrance or charge repulsion [22]. Molecular weight and conformational rigidity also play roles, as high-molecular-weight or crystalline/helix-forming mannans are less soluble than amorphous, low-molecular-weight derivatives [23]. Beyond structure, solubility is influenced by pH, temperature, ionic strength, and solvent composition of the milieu; for example, elevated temperatures or enzymatic hydrolysis enhance dissolution by reducing chain interactions [3]. Overall, mannans are most soluble when structurally branched, moderately substituted, and subjected to favorable physicochemical conditions.

### 3.2. Viscosity and Rheology

Mannans, particularly galactomannans, form highly viscous solutions even at low concentrations, making them effective stabilizers and thickeners in food and pharmaceutical formulations [24]. Their viscosity depends on molecular weight, branching, and solution conditions such as pH and ionic strength [25]. For instance, guar gum, with a high galactose substitution, exhibits superior hydration and viscosity compared to LBG, which has lower galactose content [13]. KGM, valued for its viscoelasticity and health benefits, is widely applied in food processing and pharmaceuticals. Yet, the relationship between KGM interactions with other polysaccharides and their effects on processing properties and health outcomes remains unclear. A review addresses this gap by analyzing recent studies on KGM-based binary thermodynamic compatibility systems, classified by fundamental gel models. Strategies for tailoring gel properties and their impacts on food production, nutrient digestion, and health are highlighted. Future food development should integrate structural food theory and colloidal nutrition within the framework of soft matter physics, chemistry, biology, and engineering to drive innovation [26]. Salecan, a novel food polysaccharide, was combined with deacetylated KGM to fabricate multifunctional hydrogels (KGSs) via scalable thermal crosslinking. This overcame konjac’s poor mechanics and processing issues. The hydrogels showed distinct viscoelasticity, shear thinning, self-recovery, and creep behaviors: KGSE had the lowest activation energy (25.9 kJ/mol), KGSD the highest creep compliance (1.003 1/Pa) and freezing resistance (−19.8 °C), and KGSA the best recovery (71.4%). As the first study on Salecan/Konjac biogels, this work demonstrates their low-temperature resistance, easy processability, and self-recovery, with potential in food, preservation, and biomedical applications [27]. More recently, guar gum as a high-viscosity dietary fibers showed improved gut integrity and microbial community in high-fat diet-fed mice [28]. Therefore, mannans, especially galactomannans and KGM, are valuable for their viscosity-driven functions in food and biomedical applications. Yet, how their interactions with other polysaccharides translate into processing and health effects remains poorly understood. Addressing these gaps through integrative approaches in soft matter science and nutrition will be key to developing next-generation functional foods and biomaterials.

### 3.3. Gelling Properties

The ability of mannans to form gels is another critical property, particularly in mixtures with other polysaccharides. Galactomannans synergistically interact with xanthan gum or carrageenan to form strong, elastic gels with enhanced water retention [29]. The gelling capacity is exploited in the food industry for texture modification and in biomedical fields for hydrogel-based drug delivery [9]. Polysaccharide-based gels, increasingly recognized alongside traditional protein gels, play key roles in Southeast Asian foods (e.g., liangfen, konjac tofu, grass jelly) and involve pectin, tamarind seed xyloglucan, KGM, and *Mesona chinensis* polysaccharide. Their gelling mechanisms support applications in food, delivery systems, tissue engineering, wound dressings, and adsorption. Future directions focus on enhancing flexibility, creating composite systems, and developing stimuli-responsive hydrogels comparable to synthetic gels [30]. A pH-responsive hydrogel (FA/β-CD@CG-HA-Zn) made from cationic guar gum and hyaluronic acid, with Zn^2+^ and ferulic acid/β-cyclodextrin complexes, adapts to irregular wounds and enables pH-triggered drug release. A fully plant-based hydrogel combining TEMPO-oxidized cellulose nanofibrils and Aloe vera polysaccharides exhibited high viscoelasticity, low shrinkage, and improved strength (2.7–13.2 kPa). Structural analyses showed synergistic interactions with cellulose, providing rigidity, and Aloe vera, adding flexibility, offering a sustainable alternative for biomedical use [31]. Therefore, mannans and their derivatives serve as versatile gelling agents whose synergistic interactions with other polysaccharides underpin applications from traditional foods to advanced biomedical hydrogels. While recent studies highlight their potential in drug delivery, wound healing, and sustainable biomaterials, further research is needed to optimize gel flexibility, composite design, and stimulus responsiveness to fully match or surpass synthetic counterparts.

Mannans exhibit diverse gelling behaviors depending on their structural features and interactions with other polysaccharides. Galactomannans such as guar gum and LBG do not form strong gels on their own but display synergistic gelation when combined with xanthan gum, carrageenan, or agar, where the degree of galactose substitution determines solubility and gel strength [13,25]. KGM, in contrast, can form thermally irreversible gels after alkali treatment, as deacetylation promotes intermolecular aggregation into a three-dimensional network [32]. These synergistic and self-gelling properties support their wide applications in food systems, where mannans act as stabilizers and texture modifiers, and in biomedical hydrogels for controlled drug release and wound healing (*vide infra*) [26,27].

### 3.4. Biodegradability and Biocompatibility

Like other polysaccharides, mannans are biodegradable and biocompatible, making them attractive as sustainable biomaterials. They are degraded by endo-β-mannanases, β-mannosidases, and α-galactosidases, producing oligosaccharides that are non-toxic and often exhibit prebiotic and immunomodulatory properties [4]. Acetylated mannans, such as softwood galactoglucomannans, exhibit reduced enzymatic accessibility, but controlled deacetylation improves biodegradability and processing potential [12].

Mannan, a major hemicellulose component, is widely found in plants. β-Mannanase, the main mannan-degrading enzyme, cleaves β-1,4-linked mannosidic bonds and is largely produced by microorganisms. With broad pH and temperature tolerance, microbial β-mannanases have applications in pharmaceuticals, feed, paper pulping, and biorefineries. Other review summarizes their origin, classification, properties, modification, immobilization, and applications, and outlines future research directions [33]. Self-assembled mannan nanogels are designed for targeted therapeutic and vaccine delivery via mannose receptors on antigen-presenting cells [34]. Studies show that in human plasma, a specific protein corona, mainly apolipoproteins B-100, A-I, E, and albumin, forms slowly around the nanogels with minimal structural changes. Mannan nanogels do not affect blood coagulation and slow fibril formation of amyloidogenic proteins. These findings demonstrate the high biocompatibility and biosafety of these nanomaterials, supporting their potential use in biomedical applications and providing a molecular framework for assessing nanomaterial safety. Mannan, along with other edible polysaccharides, is used in protein-polysaccharide nanoconjugates as a biocompatible and biodegradable building block for the nanoencapsulation of nutraceuticals [35]. These mannan-based nanostructures enhance the stability, delivery, and bioavailability of plant-derived bioactive compounds in food applications, leveraging their physicochemical properties and ability to form covalent networks with proteins such as gelatin, soy, or zein. This highlights the potential of mannan-containing nanoconjugates as functional delivery systems in food nanotechnology. Porous microgels loaded with MOS offer a drug-free, bioinspired strategy for managing inflammatory bowel disease (IBD) by mimicking intestinal cell binding sites [36]. These microgels attract adherent-invasive *Escherichia coli* via FimH, preventing gut colonization. In a mouse model, MOS microgels reduce inflammation, decrease harmful bacteria, and enhance microbial diversity, demonstrating a non-antibiotic approach to restore gut microbial balance.

### 3.5. Functional Modifications

Chemical modifications, such as acetylation, carboxymethylation, and sulfation, can alter the solubility, charge distribution, and bioactivity of mannan. Sulfated mannans from marine algae, for example, display unique physicochemical profiles that enable anticoagulant and antiviral activities [22]. These modifications expand their potential applications in biomedical, food, and pharmaceutical sectors. Acetylation modifies water solubility and enzymatic accessibility, impacting their biodegradability and industrial processing of GGM [3].

In summary, the physicochemical versatility of mannans, from insoluble structural polymers to highly soluble hydrocolloids, underlies their widespread use as functional biopolymers in diverse industries [37]. Their tunable properties make them particularly valuable as renewable, biodegradable, and biocompatible materials for emerging biotechnological applications (vide infra).

## 4. Natural Sources of Mannans

**Figure 1:**
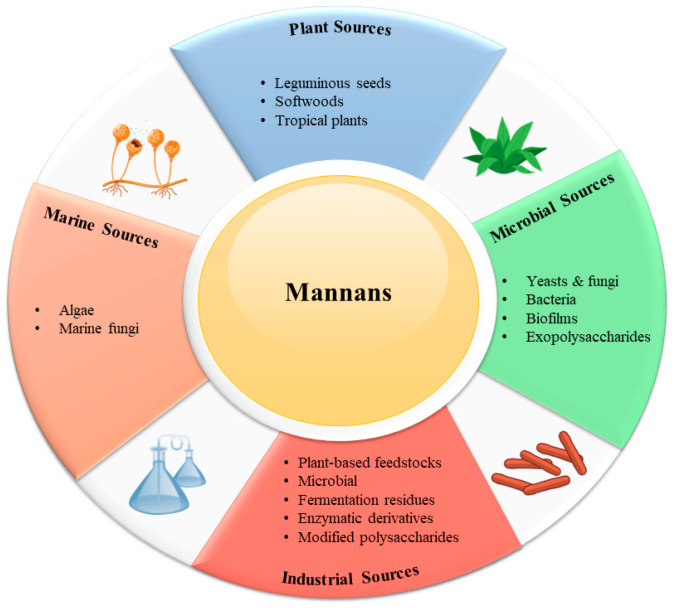
Distinguished resources of mannans.

Mannans are widely distributed in nature, occurring in plants, microorganisms, and marine organisms, where they serve both structural and functional roles. Their abundance and diversity of sources make them an important class of renewable biopolymers (Figure 1).

### 4.1. Plant Sources

In higher plants, mannans are prominent constituents of the hemicellulosic fraction of cell walls, especially in seeds and woody tissues. Leguminous seeds such as guar (*Cyamopsis tetragonoloba*) and locust bean (*Ceratonia siliqua*) are rich in galactomannans, which accumulate in the endosperm as reserve carbohydrates for seed germination [4,12]. In softwoods (gymnosperms), glucomannans and GGMs account for up to 20% of dry weight, playing a vital role in secondary wall architecture and mechanical stability [38]. Tropical plants such as ivory nuts contain linear mannans, which represent relatively rare homopolymers in nature [3]. Galactomannans from Gleditsia fruit seeds (GSGs) are extracted using hot water, microwave, and ultrasonic techniques, showing molecular weights from 0.018 × 10^3^ to 2.778 × 10^3^ kDa with mannose, galactose, glucose, and arabinose as main constituents. They demonstrate strong antioxidant, hypoglycemic, hypolipidemic, and anti-inflammatory activities with high bioavailability, biocompatibility, and biodegradability. Owing to these properties, GSGs hold promising applications in food, pharmaceuticals, packaging, and agriculture, although further research is needed to overcome current limitations [39].

Mannan, a key polysaccharide in softwoods and other plants, varies widely in composition and structure. Its complete hydrolysis requires multiple enzymes acting synergistically. Studies show that supplementing β-mannanases with β-mannosidases or α-galactosidases can enhance hydrolysis, though effects differ: mannosidases may compete with mannanases, while α-galactosidases from different families display varying synergistic behaviors. A deeper understanding of these interactions is crucial for improving sugar yields and optimizing enzyme mixtures. A seminal review highlights current knowledge gaps and their relevance to efficient conversion of mannan-rich biomass, such as softwoods, into fermentable sugars for bioethanol production [40].

Next-generation biomaterials seek to unite mechanical strength with biological function. Plant-derived glucomannans such as KGM and *Bletilla striata* polysaccharide (BSP) show promise for drug delivery and wound dressings due to their gelling ability, biocompatibility, modifiability, and bioactivities, underscoring their biomedical potential [41,42]. Other study assessed pressurized hot water extracts from Norway spruce bark and birch sawdust, which were rich in galactoglucomannan and glucuronoxylan. The extracts protected erythrocytes, showed antidiabetic activity, and were non-toxic to human cells, highlighting their promise as safe, sustainable food ingredients [43]. In summary, mannan-based polymers and their combinatorial pharmaceutical formulations represent an emerging research frontier, with a new generation of products expected to reach the market in the near future.

### 4.2. Microbial Sources

The interplay between mannans and microbes was first highlighted in seminal studies revealing that immune lectins can specifically recognize and bind mannans (for a review, see [44]. Mannans are integral components of fungal and yeast cell walls, where they are covalently linked to proteins forming mannoproteins. In *Saccharomyces cerevisiae* and pathogenic fungi such as *Candida albicans*, cell wall mannans are recognized by host immune receptors, making them crucial in pathogenesis and host–pathogen interactions [6]. Mannans derived from *S. cerevisiae* and other yeasts exhibit diverse bioactivities, including immunomodulatory, antioxidative, antiviral, and gut microbiota-modulating effects. Their structure-dependent functionalities position them as promising sustainable ingredients for food, feed, and pharmaceutical applications [45]. Hemicellulose is a diverse group of polysaccharides, and their degradation requires multiple hemicellulases. Beyond traditional Trichoderma and Aspergillus sources, Penicillium and Talaromyces have emerged as promising producers. Their enzymes offer new opportunities for lignocellulosic biomass valorization in a circular economy (for a review, see [46]). The opportunistic yeast *Cryptococcus neoformans* has a polysaccharide capsule, a key virulence factor. Notably, mannans play a central role in capsule architecture: one major component is a mannan polymer substituted with xylose and glucuronic acid, while a second polysaccharide, a galactan, carries galactomannan side chains decorated with varying amounts of these sugars. Mannoproteins within the underlying cell wall further emphasize the importance of mannans in the structural integrity and host interactions of *C. neoformans*. Advances in this field have revealed how mannan-rich structures reinforce the biology and pathogenicity of *C. neoformans*, and suggest new opportunities to target mannan synthesis and organization in antifungal strategies (for a review, see [47]). In sum, many microbes express enzymes for the biosynthesis and biodegradation of mannans, and many researchers are involved in this case. For example, *Debaryomyces hansenii* Y4 possesses mannosidase [48]. Research on antibiotic alternatives has highlighted yeast cell wall β-glucan and mannan as promising substitutes for preventing and treating animal diseases, helping curb antibiotic resistance. These polysaccharides enhance gut health, immunity, antioxidant capacity, and mycotoxin adsorption, with their activities further improved by structural modification or molecular weight reduction [49]. In sum, mannans are multifunctional polysaccharides central to microbial physiology, host–pathogen interactions, and biotechnological applications. They serve as structural components in fungal and yeast cell walls, contribute to virulence in pathogens like *Cryptococcus neoformans*, and act as immunomodulatory and bioactive compounds in food, feed, and pharmaceuticals. Microbial enzymes both synthesize and degrade mannans, enabling biomass valorization and supporting alternatives to antibiotics, while structural modifications of mannans further enhance their functional properties.

Certain bacteria also produce extracellular mannans, often contributing to biofilm formation and immune evasion [50]. One study provides the first proteomic analysis of *Chitinophaga pinensis*’s biomass-degrading machinery, with a focus on mannan, a major plant hemicellulose. Several novel mannan-degrading enzymes were identified, along with substrate-specific Polysaccharide Utilization Loci, revealing how the bacterium targets mannan and other biomass components for nutrient acquisition. These findings highlight mannan-active enzymes as promising candidates for future biotechnological applications [51]. Other study analyzed the structure and properties of a mannan exopolysaccharide from the cold-adapted bacterium *Psychrobacter arcticus* [18]. Therefore, mannans play dual roles in both microbial physiology and biotechnology as structural and functional components in bacterial biofilms and as substrates for novel mannan-degrading enzymes, while their exopolysaccharide forms from cold-adapted bacteria reveal unique structural features with potential functional applications.

### 4.3. Marine Sources

Marine organisms provide unique mannans, particularly sulfated mannans isolated from red and green algae. These structurally modified mannans exhibit bioactive properties, including anticoagulant, antiviral, and immunomodulatory activities, making them promising candidates for biomedical exploitation [22]. Brown algae, while dominated by alginates, also contain mannan derivatives contributing to their cell wall complexity [52].Marine-derived mannans, particularly sulfated forms, exhibit broad-spectrum antiviral activity against viruses such as HIV, HSV, influenza, hepatitis C, and dengue. Their mechanisms involve blocking viral attachment, entry, or replication, often through sulfate-mediated interactions. These structurally diverse marine mannans hold significant promise as leads for developing next-generation antiviral therapeutics [53]. Marine green algae are rich sources of structurally diverse sulfated polysaccharides, including ulvans, rhamnans, arabinogalactans, galactans, and sulfated mannans. These compounds exhibit broad bioactivities, including anticoagulant, antiviral, antioxidative, antitumor, immunomodulatory, antihyperlipidemic, and hepatoprotective, highlighting their potential for nutraceutical and medical applications [54]. Sulfated polysaccharides from the green alga *Codium fragile* are particularly noteworthy for their unique structures, pyruvylated β-d-galactan sulfates, sulfated arabinogalactans, sulfated β-l-arabinans, and sulfated β-d-mannans, and broad bioactivities, including anticoagulant, immune-enhancing, anticancer, antioxidant, and drug-delivery functions. One review highlights the structural and functional diversity of Codium sulfated polysaccharides and addresses strategies to overcome industrial challenges in production, purification, and quality control [55]. A mannan (HPA) from the marine fungus *Hansfordia sinuosae* alleviated ulcerative colitis in mice. HPA reduced inflammation by suppressing NLRP3, TNF-α, and IL-1β, increased IL-10, repaired the colonic barrier, and restored gut microbiota balance by promoting beneficial bacteria like Bifidobacterium. Additionally, HPA mitigated depression-like behavior, highlighting its potential as a mannose-based therapeutic or functional food for colitis and related mood disorders [27]. In sum, marine-derived mannans, particularly sulfated forms from red and green algae and mannans from marine fungi, exhibit diverse bioactivities, including antiviral, anticoagulant, immunomodulatory, antioxidant, anticancer, and gut-protective effects. Their structural diversity, such as sulfation or pyruvylation, underlies these functions, making them promising candidates for biomedical, nutraceutical, and therapeutic applications. Mannose-based marine polysaccharides, like HPA from *Hansfordia sinuosae*, further demonstrate potential in treating inflammatory disorders and modulating gut microbiota.

### 4.4. Industrial Sources

Commercially, mannans are primarily extracted from leguminous seeds (such as guar gum and LBG) and softwood pulping residues (galactoglucomannans) [8,38]. These sources represent scalable and cost-effective feedstocks, enabling their use in food, pharmaceuticals, the paper industry, and biomaterial applications. Microbial mannanases are biotechnologically important enzymes that hydrolyze complex plant polysaccharides into simple sugars such as manno-oligosaccharides and mannose. Widely applied in the paper and pulp industry, they are now also used in food, feed, coffee extraction, oil drilling, and detergents. Mannan, the main hemicellulose in softwoods, is hydrolyzed by β-mannosidases, exo-acting enzymes that release mannose from mannooligomers and mannobiose. These enzymes are produced mainly by bacteria and fungi, and these enzymes are often extracellular and active across broad pH and temperature ranges. They have applications in bioethanol production, alkyl glycoside synthesis, and pharmaceuticals. One review summarizes microbial mannosidases with emphasis on their sources, production, properties, cloning, and biotechnological uses [56]. While mannanases are produced by microorganisms, plants and animals also contribute. Bacterial mannanases are mainly extracellular and active across broad pH and temperature ranges, with acidic and neutral forms being most common. Other review emphasizes microbial mannanases, covering mannan structure, enzyme complexes for its degradation, sources, production, and industrial applications [57].

Mannans are widely distributed in plants. Their complete degradation requires β-mannanases, β-mannosidases, β-glucosidases, and accessory enzymes such as α-galactosidases and acetyl mannan esterases. Mannanases are produced by bacteria, fungi, actinomycetes, plants, and animals, with microbial enzymes, mostly extracellular and active across broad pH and temperature ranges, being the most industrially relevant. Owing to these properties, microbial mannanases are applied in pulp and paper, pharmaceuticals, food, feed, oil, and textiles. Other review highlights recent advances in microbial sources, production, enzyme properties, heterologous expression, and industrial applications [58]. Renewable biomaterials and functionalized biopolymers are gaining industrial importance as sustainable alternatives to fossil-based polymers. Plant polysaccharides, in natural or modified forms, are widely applied in biomedical, food, feed, and packaging sectors. Enzymatic modifications further expand their potential. An impressive review summarizes recent advances in the enzymatic oxidation of galactomannans from leguminous plants, with emphasis on the versatile laccase/TEMPO system. This reaction induces polymer cross-linking, transforming galactomannan solutions into elastic gels that, upon lyophilization, yield stable aerogels. Such aerogels show promise as delivery systems for bioactive agents, demonstrated with antibiotics, antimicrobial peptides, enzymes, and industrial microbiocides [59].

Sustainable industry practices promote converting production waste into valuable by-products. Spent *Saccharomyces cerevisiae* from fermentation, often used as animal feed or discarded, can instead provide mannans and MOS. Other review summarizes chemical, enzymatic, and physical methods for extracting mannans and producing MOS, as well as chemical modifications to enhance their properties. Key bioactivities, potential applications, and commercially available products containing mannans, MOS, and mannose are also discussed [60]. β-Mannooligosaccharides (β-MOS) are emerging prebiotics derived from β-mannan that selectively promote beneficial gut microbiota and produce health-promoting metabolites like short-chain fatty acids. Enzymatic production using β-mannanases is the most efficient and eco-friendly method. Still, large-scale application requires optimized production conditions, low-cost substrates, and further in vivo and clinical validation. One review summarizes β-MOS production, characterization, bioactivities, structural-functional relationships, and in vivo studies, highlighting research gaps and future prospects for their use as prebiotics, functional foods, and therapeutic agents [61]. Overall, the diverse sources of mannans reflect their evolutionary conservation and functional versatility, ranging from structural components in plants to immune-modulating molecules in microbes and algae. This diversity also broadens their potential as sustainable biopolymers for industrial and biomedical applications.

## 5. Biological Functions and Bioactivities

**Figure 2:**
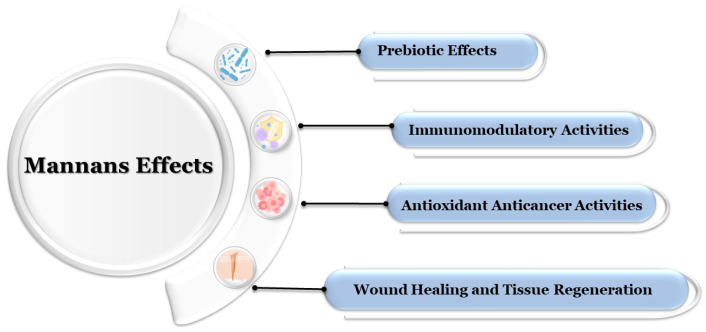
Various effects of mannans in biomedical science.

Mannans are not only structural polysaccharides but also exhibit a broad spectrum of biological activities, making them valuable in both natural ecosystems and biomedical applications (Figure 2). Their bioactivity is determined by their molecular structure, degree of branching, and chemical modifications [4,9].

### 5.1. Prebiotic Effects

MOS are widely recognized as prebiotics that resist digestion in the upper gastrointestinal tract and are fermented in the colon, selectively stimulating beneficial microbes such as *Bifidobacterium* and *Lactobacillus* species [62]. This fermentation produces short-chain fatty acids that support gut health and immune regulation, leading to the incorporation of MOS into functional foods and animal feeds to enhance performance and disease resistance. Yeast-derived mannoproteins also hold promise as bioactive agents for probiotic delivery by improving adhesion, survival, and prebiotic effects, with optimized extraction methods enabling their use in synbiotic formulations [63]. In parallel, natural gums such as gum Arabic, guar gum, and xanthan gum have been evaluated as prebiotic sources for synbiotic foods, though challenges, including microbial degradation, viscosity loss, and solubility remain [64].

Beyond traditional food applications, mannans and their derivatives exhibit broad biomedical potential. MOS produced enzymatically from agro-residues show advantages over chemical methods and are already commercialized from yeast cell walls for poultry and aquaculture feeds [65]. KGM has demonstrated prebiotic, anti-inflammatory, and antitumor properties [66]. In neurological contexts, MOS improved cognition, reduced amyloid accumulation, and preserved gut integrity in mouse models of Alzheimer’s disease [49]. Similarly, yeast α-mannans support beneficial microbial fermentation patterns [67], while different mannan substrates such as guar, locust bean, and konjac show distinct effects on short-chain fatty acid production and microbial shifts [68]. Advances in metabolic engineering have further enhanced mannan content in *Saccharomyces boulardii*, improving its adhesion and selective probiotic-promoting capacity [69].

The prebiotic potential of mannans extends into diverse sources and applications. Mushroom-derived mannans and related polysaccharides support probiotic growth while offering antioxidative and medicinal benefits [70]. In the gut, Bacteroidota and Bacillota exploit mannan diversity using specialized enzymes and transport systems, linking dietary mannans to microbial foraging strategies and host metabolism [71]. In animal production, synbiotics combining MOS with probiotics improved growth and health in poultry [72], while mannan-rich fractions reduced pathogenic microbes and antimicrobial resistance genes in laying hens [73]. In aquaculture, dietary MOS enhanced growth, immunity, and stress resilience in tilapia [74]. Collectively, mannans and their derivatives function as versatile prebiotics with applications across food, biomedical, and animal health sectors. Their structural diversity enables targeted modulation of gut microbiota, immune responses, and even neuroprotection, while ongoing innovations in extraction, fermentation, and metabolic engineering continue to expand their functional potential.

### 5.2. Immunomodulatory Activities

Mannans and mannan-derived oligosaccharides exert strong immunomodulatory effects by interacting with pattern recognition receptors such as mannose receptors, Toll-like receptors, and Dectin-2 on immune cells [75]. Yeast-derived mannans activate macrophages, stimulate cytokine secretion, and enhance antigen presentation, thereby bridging innate and adaptive immunity [6]. Recently, Portuguese scientists showed that mannan can trigger an enhanced immune phenotype compatible with trained immunity in healthy monocytes, with glycan-primed cells exhibiting enhanced pro-inflammatory cytokine secretion and higher activation (CD86) levels that terminated towards an effective anti-tumor immune response [76]. In this line, PI-88, a sulfonated derivative of yeast phosphomannan, is under clinical evaluation as an anticancer agent [41]. Sulfated mannans from algae further demonstrate anti-inflammatory activity through modulating nitric oxide and cytokine production [22]. Their diverse immunological roles underscore their potential in regulating host defense and inflammation. Clinical and molecular studies highlight additional complexities of mannan–immune system interactions. Anti-*Saccharomyces cerevisiae* antibodies, which target yeast mannans, are widely used to differentiate Crohn’s disease from ulcerative colitis, yet their heterogeneity, generation, and persistence remain unresolved [77]. Beyond natural immune responses, mannan-based glycomimetics are being developed to mimic fungal mannans and target phosphodiester linkages critical for eliciting antifungal immunity against pathogens like *Candida albicans* and *Candida auris* [78]. Together, these findings position mannans as both natural immune modulators and engineered immunotherapeutic platforms.

Applications of mannans in biomedicine continue to expand. KGM, with strong water retention and gel-forming properties, can be chemically modified to enhance solubility, viscosity, and mechanical strength, while regulating macrophage polarization in contexts such as wound healing, IBD, cancer therapy, and vaccine delivery [79]. Conjugating mannans with protein antigens, such as the *Mycobacterium tuberculosis* fusion protein, has significantly enhanced vaccine immunogenicity, driving robust cytokine responses, T cell proliferation, and antibody production [41]. Similarly, in aquaculture, mannans have been shown to induce trained immunity in pufferfish, elevating oxidative and metabolic responses that improve resistance to bacterial pathogens [80]. In sum, mannans act as versatile immunomodulators capable of enhancing innate memory, shaping adaptive immunity, and serving as adjuvants or structural platforms in vaccines and therapeutics. Their ability to influence both host and pathogen interactions underscores their promise as multifunctional agents in human health, veterinary applications, and immunotherapy.

### 5.3. Antimicrobial Effects

Mannans and their derivatives exhibit notable antiviral activities, primarily by functioning as immunomodulatory adjuvants and viral entry inhibitors. Sulfated mannans from marine algae can block viral adsorption and replication while enhancing host antiviral immunity [81,82]. Oxidized mannans have been tested as vaccine adjuvants, significantly improving immune responses in veterinary rabies vaccines [83]. Similarly, D-galacto-D-mannan has been identified as a potent Dectin-2 agonist that boosts both humoral and cellular immune responses in foot-and-mouth disease vaccines in mice and pigs [84]. Mannan-based adjuvants are increasingly incorporated into vaccine formulations to enhance antigen presentation, cytokine production, and antibody generation, positioning them as promising candidates for next-generation antiviral strategies [84,85].

Mannans and MOS exhibit notable antibacterial effects through multiple mechanisms, including blocking pathogen adhesion, modulating host immunity, and reshaping the gut microbiota. Yeast-derived MOS can bind to type-1 fimbriae of enteric pathogens such as *Escherichia coli* and *Salmonella enterica*, thereby preventing their colonization of the intestinal epithelium [86]. In poultry and livestock, dietary supplementation with MOS has been shown to reduce pathogenic bacterial loads, particularly *Salmonella* and *Clostridium perfringens*, while simultaneously enriching beneficial *Lactobacillus* and *Bifidobacterium* populations [73,87]. Mannan-rich fractions from yeast cell walls also attenuate the abundance of antimicrobial resistance-associated pathogens, including *Escherichia* and *Brachyspira*, supporting their role as sustainable alternatives for antibiotics [73]. In aquaculture, MOS supplementation improve disease resistance in fish by reducing Aeromonas and Vibrio infections, alongside enhancing immune gene expression and gut health [74]. Together, these findings highlight mannans as multifunctional antibacterial agents with applications in food safety, animal health, and microbiome modulation.

Mannans, particularly those derived from yeast and fungi, display significant antifungal properties by modulating host immune responses and interfering with fungal cell wall recognition. Yeast mannans act as PAMPs that are recognized by pattern recognition receptors such as Dectin-2, mannose receptor, and Toll-like receptors, leading to macrophage activation, cytokine release, and enhanced antifungal immunity [6]. ASCA which target yeast mannans, have been used as biomarkers for fungal-associated immune disorders and highlight the immunogenicity of mannans in antifungal responses [77]. Moreover, synthetic mannan glycomimetics designed to mimic fungal mannans have emerged as promising candidates for vaccine adjuvants, enhancing immune recognition and response against pathogenic fungi such as *Candida albicans* and *Candida auris* [78]. Sulfated mannans from marine algae also demonstrate direct antifungal effects by inhibiting fungal growth and reducing inflammatory damage, further expanding their therapeutic potential [22]. Collectively, mannans function both as immune modulators and direct antifungal agents, making them valuable for antifungal therapy and vaccine development.

Direct evidence that mannans themselves kill helminths is scarce, while most investigations support points to indirect anthelmintic effects, immune modulation, microbiome shifts, and barrier enhancement, rather than a classical vermicidal action. Mannans have little direct, well-documented vermicidal activity, but they can reduce helminth burden indirectly by strengthening host defenses and altering the gut environment. In this line, mannose-recognition pathways (e.g., the mannose receptor) shape host immune responses to helminths and can promote anti-inflammatory or protective programs [88]. Dietary mannan-rich yeast fractions and MOS modify gut barrier function and the microbiome, which in some animal studies is associated with reduced susceptibility to ecto- or endo-parasites or improved host resistance [89,90]. Review of glycoconjugates in host–helminth interactions emphasizes that helminths and host immune systems use mannans and related glycans as signals, but they do not report mannans as direct anthelmintics [91]. In sum, current evidence supports immune-mediated and microbiome-mediated anthelmintic effects of mannan preparations rather than direct worm-killing activity. Targeted studies testing purified mannans and defined MOS against helminths in vitro and in vivo are needed to settle the question.

### 5.4. Antioxidative and Anticancer Activities

Mannans and MOS have been increasingly recognized for their antioxidative properties, which complement their prebiotic and immunomodulatory roles [92]. Acemannan, a bioactive acetylated mannan derived from Aloe vera, has been extensively studied for its potential anticancer effects, which include enhancing immune responses against tumors and improving the efficacy of chemotherapeutic agents [93]. Its ability to stimulate macrophages and T-lymphocytes makes it a promising candidate in cancer immunotherapy. Yeast-derived mannans can scavenge free radicals and reduce oxidative stress through enhancing endogenous antioxidant enzyme activities such as superoxide dismutase, catalase, and glutathione peroxidase [94]. Sulfated mannans from marine algae exhibit even stronger antioxidative activity, attributed to their high sulfate substitution, which enhances hydrogen-donating ability and metal-chelating capacity [95]. MOS have also been reported to lower malondialdehyde levels and restore redox balance in vivo, suggesting protective effects against lipid peroxidation and oxidative damage [49]. These findings highlight mannans as promising natural antioxidants, with potential applications in functional foods, nutraceuticals, and biomedical formulations aimed at mitigating oxidative stress-related disorders.

Mannans and their derivatives have demonstrated anticancer potential through multiple mechanisms, including immune modulation, apoptosis induction, and inhibition of tumor progression. Yeast-derived mannans, particularly mannoproteins, can activate macrophages and dendritic cells via mannose receptors and Toll-like receptors, leading to cytokine secretion (e.g., TNF-α, IL-6) and stimulation of antitumor immune responses [6]. Sulfated mannans from marine algae exhibit strong anticancer activity by inducing cell cycle arrest and apoptosis, partly attributed to their ability to modulate oxidative stress and inhibit tumor angiogenesis [96]. KGM and its derivatives also show promise as anticancer agents. The modified KGM enhances drug delivery efficiency, promotes apoptosis in cancer cells, and reduces tumor growth in vivo [79]. Additionally, mannan-based vaccine adjuvants, such as mannan–antigen conjugates, have been developed to boost antitumor immunity by enhancing antigen presentation and T-cell activation [41]. Other study evaluated Aloe vera polysaccharides (fractions A50 and I50) for their anticancer potential. Both fractions modulated phthalate-induced glycosylation changes, with A50 reducing cell viability in a dose-dependent manner, while I50 more effectively inhibited migration, invasion, and stemness. These distinct effects, likely due to structural differences, suggest Aloe vera polysaccharides as promising candidates for anticancer therapy and warrant further clinical investigation [97]. Collectively, these findings highlight mannans as multifunctional candidates for anticancer strategies, both as direct bioactives and as carriers/adjuvants in immunotherapy.

### 5.5. Wound Healing and Tissue Regeneration

Mannans have emerged as promising biomaterials for wound healing and tissue regeneration due to their biocompatibility, biodegradability, and ability to modulate immune and cellular responses. Plant-derived mannans, such as KGM, are valued for their water retention, viscosity, and gel-forming capacity, which support moist wound environments and promote cell proliferation. Chemical modifications of KGM (e.g., oxidation, acetylation, and cationization) further enhance its mechanical strength, bioactivity, and ability to regulate macrophage polarization, thereby accelerating wound repair and reducing inflammation [79]. Yeast-derived mannans and mannoproteins act as immunomodulators by stimulating macrophages and dendritic cells, enhancing cytokine production, and promoting angiogenesis, which are essential for tissue regeneration [6]. Additionally, mannose-functionalized hydrogels and nanogels have been engineered as drug-delivery dressings for wounds, enabling controlled release of antimicrobials, growth factors, or anti-inflammatory agents, while supporting vascularization and re-epithelialization [27]. Collectively, these properties highlight mannans as multifunctional candidates for next-generation wound dressings and regenerative biomaterials. Bioactive mannans such as acemannan also promote wound healing by stimulating fibroblast proliferation, collagen deposition, and angiogenesis [98]. Their biocompatibility, biodegradability, and ability to form hydrogels make them suitable as scaffolds in tissue engineering and regenerative medicine [9]. One study explored acemannan-coated, cobalt-doped biphasic calcium phosphate nanoparticles for bone regeneration. The nanoparticles promoted proliferation, osteogenic differentiation, and calcium deposition without altering MC3T3-E1 morphology. They reduced M1 markers (iNOS, CD68) and enhanced M2 markers (CD206, CD163, Arg-1), creating a pro-healing immune microenvironment. Findings demonstrate, for the first time, that combining acemannan and cobalt supports osteogenesis via immunomodulation, offering a novel strategy for bone repair [99]. Therefore, mannans are multifunctional biomaterials that support wound healing and tissue regeneration by enhancing fibroblast growth, collagen deposition, angiogenesis, and immune modulation. From natural sources like glucomannan and acemannan to engineered hydrogels and nanocomposites, they offer strong potential for next-generation dressings and regenerative scaffolds.

## 6. Industrial and Biomedical Applications of Mannans

**Figure 3:**
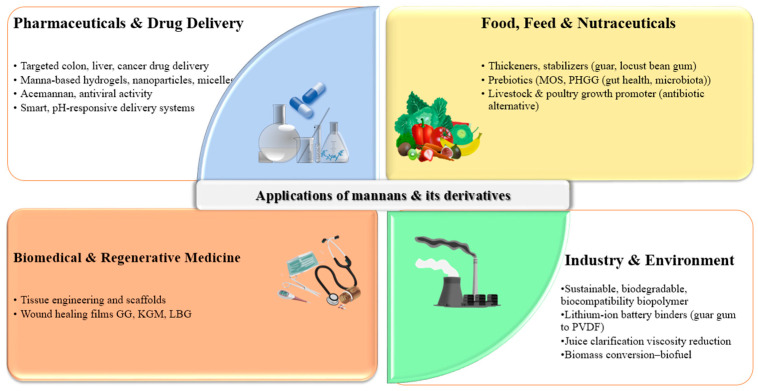
Mannans at the intercept of various industries. GG: Guar Gum; KGM: Konjac Glucomannan; LBG: Locust Bean Gum; MOS: Mannan Oligosaccharides; PHGG: Partially Hydrolyzed Guar Gum; PVDF: Polyvinylidene Fluoride.

Due to their unique structural diversity, physicochemical versatility, and biological activities, mannans and their derivatives have found widespread applications in food, pharmaceutical, biomedical, and environmental sectors (Figure 3). Their biodegradability, non-toxicity, and biocompatibility further strengthen their role as sustainable biopolymers for industrial innovation [9,12]. For example, lithium-ion batteries (LIBs) have become essential for portable electronics and are now critical for hybrid and electric vehicles since the 1990s. Despite their high energy density, LIBs face challenges such as cost, safety risks, and limitations of polyvinylidene fluoride binders, which are costly, hard to recycle, and unstable at high temperatures. A mini-review highlights guar gum as an alternative binder for electrodes and separators, comparing its electrochemical performance with that of conventional binders [100]. Industrial applications extend further to mannan-degrading enzymes which are employed in juice clarification, viscosity reduction, and biomass conversion for biofuels, adding economic and environmental value to agricultural and forestry residues [3,101]. Due to their high abundance, glucomannans are considered potential feedstocks for biofuel and biopolymer production [102]. Here, we discuss the applications of mannans and their derivatives in various industrial sectors (vide infra).

### 6.1. Food, Feed, and Nutraceutical Industry

Mannans and their derivatives, including galactomannans, glucomannans, and MOS, have diverse applications across food, feed, and nutraceutical sectors. In foods, galactomannans such as guar gum and LBG act as thickeners, stabilizers, and emulsifiers, improving texture, shelf-life, and mouthfeel under different processing conditions [8,32,101]. KGM is widely used for gelation and thickening, with nutritional and functional benefits linked to viscosity, solubility, and deacetylation [103]. Beyond food use, mannans derived from wood and other biomass represent underexploited but sustainable resources with potential as novel dietary fibers and functional ingredients [104]. In human nutrition, MOS and partially hydrolyzed guar gum (PHGG) have shown clinical benefits as prebiotics. PHGG improved stool form and increased Bifidobacterium abundance in irritable bowel syndrome patients [105], while a fiber supplement containing KGM, galacto-oligosaccharides, and Bifidobacterium exopolysaccharides reduced HbA1c and fasting glucose in prediabetic individuals through microbiota modulation [106].

In livestock and poultry, MOS are well established as alternatives to antibiotic growth promoters. In poultry, MOS supplementation improved carcass traits, reduced fat and cholesterol, enhanced antioxidative activity, and supported gut integrity [107]. In swine, MOS improved immune responses, nutrient utilization, and gut metabolites, with β-mannanase supplementation enhancing growth and feed efficiency, though responses vary by age and production stage [15,108,109]. In (pseudo)ruminants, MOS improved growth, feed efficiency, and disease resistance, with benefits observed in calves [110], beef cattle [111], and rabbits, where MOS enhanced gut health and productivity [112]. In aquaculture, MOS supplementation improved immune biomarkers, antimicrobial peptide expression, and resistance to bacterial infection in grass carp [113]. Canine studies also suggest MOS supplementation supports microbiota diversity, short-chain fatty acids production, and anti-inflammatory effects in IBD [114]. In summary, mannans and their derivatives function as multifunctional bioactive polysaccharides enhancing food quality, acting as prebiotics, supporting gut health, and serving as sustainable feed additives across livestock, poultry, aquaculture, and companion animals. Their versatility and sustainability highlight strong potential for future innovations in food, feed, and health applications.

### 6.2. Pharmaceutical and Drug Delivery Systems

Carbohydrate polymers are vital for targeted drug delivery, especially in colon cancer. Modifications enhance drug loading, stability, and release, improving therapeutic efficacy. Chitosan nanoparticles are pH-responsive, while pectin resists gastric enzymes, enabling colon-specific delivery. Combining these polymers with nanotechnology, 3D printing, and artificial intelligence allows stimuli-responsive systems that release drugs precisely in response to pH, redox potential, or colon enzymes, reducing toxicity and improving treatment outcomes [115]. Plant-derived hemicelluloses, including mannans, xylans, and arabinoxylans, are linear polysaccharides with high biocompatibility, biodegradability, and low immunogenicity. Their functional properties, such as cell adhesiveness and ease of chemical modification, make them ideal for tissue engineering, drug delivery, and gene delivery. Recent advances highlight their versatility and structural diversity, expanding potential biomedical applications ([9]. For instance, galactomannans, biodegradable polysaccharides from sources like guar, locust bean, and fenugreek gums, are widely studied for drug delivery systems including tablets, nanoparticles, hydrogels, and micelles. These nanomaterials show promise in oral vaccines, insulin delivery, cancer targeting, wound dressings, and heavy metal removal, with some also used in green synthesis of antimicrobial, antioxidative, and anticancer metal nanoparticles. Lesser-studied galactomannans (cassia, tara, Delonix, etc.) present additional opportunities for biomedical applications [116].

The biocompatibility and film-forming ability of mannans have led to their exploration in drug delivery systems. Mannans can be chemically modified to create targeted drug carriers, particularly for liver-specific delivery due to the affinity of mannose receptors expressed on hepatocytes and macrophages [101]. Acemannan, an acetylated mannan derived from Aloe vera, has been incorporated into oral and topical formulations due to its immunostimulatory, wound-healing, and antiviral properties [92]. Additionally, sulfated mannans have been developed as antiviral agents due to their ability to inhibit viral attachment and replication [81]. Mannans can be functionalized and copolymerized with other compounds. In this case, an Aloe vera mucilage/acrylic acid copolymer hydrogel (ALH-g-PAA) was synthesized via free radical copolymerization. Spectroscopic studies confirmed its superporous structure, with swelling and porosity. The hydrogel swelled strongly at pH 7.4, weakly at pH 1.2, and showed pH-responsive on/off switching. Metoprolol tartrate was released in a pH-, time-, and swelling-dependent manner over 24 h, fitting first-order and Korsmeyer–Peppas models. ALH-g-PAA was hemocompatible, indicating promise for sustained drug delivery [117]. Other researchers developed pH-responsive hybrid cubosomes surface-modified with chitosan-N-arginine/alginate and loaded with acemannan [118]. The particles achieved high encapsulation and controlled release under gastric and intestinal pH. Acemannan induced a phase transition, compressing nanochannels and enhancing release. The cubosomes also showed strong affinity for high-curvature membranes and induced vesicle remodeling. These smart, structure-responsive bioparticles hold promise for pH-triggered gastrointestinal drug delivery and enhanced cellular uptake.

Guar gum, a plant-derived polysaccharide, has attracted considerable attention as a natural, biocompatible, and biodegradable material for drug delivery applications. Its widespread availability, non-toxicity, eco-friendliness, and cost-effectiveness make it an ideal carrier for therapeutic agents. Guar gum-based systems, including hydrogels, films, scaffolds, nanoparticles, and nanocomposites, have been extensively studied for controlled and sustained drug release through oral, buccal, transdermal, intravenous, and gene delivery routes. Recent reviews highlight advances in guar gam-based hydrogels, nanoparticles, and scaffolds, emphasizing their drug loading capacity and release profiles that support improved targeted therapies [119,120]. Beyond drug delivery, guar gum supplementation has demonstrated health benefits; a dose–response meta-analysis of 25 clinical trials reported significant reductions in total cholesterol and low-density lipoprotein cholesterol in patients with cardiometabolic conditions, though no significant effects on triglycerides or high-density lipoprotein cholesterol were observed [121]. Guar gum is a biopolymer with pH-dependent swelling and microbial degradation in the colon, making it ideal for colon-specific drug delivery. Its uncontrolled hydration can be managed via structural modification or grafting with other polymers. Various guar gum derivatives have been developed and evaluated for colon targeting, including matrix tablets, coated formulations, nano/microparticles, and hydrogels, demonstrating their potential to localize therapeutics in the colonic environment [122].

KGM, a biodegradable and biocompatible natural polymer with strong gelation, adhesion, and film-forming properties, has been widely investigated for drug delivery applications. KGM-based composites, including gels, films, microspheres, nanofibers, and nanoparticles, demonstrate significant potential in drug delivery, wound healing, tissue engineering, antibacterial, and cancer therapies, though challenges remain in advancing their biomedical applications [123]. A stimuli-responsive hydrogel system incorporating curcumin-loaded mesoporous polydopamine nanoparticles (mPDA NPs@Cur) into oxidized KGM/carboxymethyl chitosan (oxKGM/CMCS) hydrogels enabled pH-sensitive and near-infrared-triggered release, providing controlled delivery and effective chemo-photothermal therapy against breast cancer cells, with curcumin release following zero-order kinetics [124]. pH-responsive bone tissue engineering microspheres prepared from KGM, hydroxyapatite (HA), and sodium alginate (SA), with quaternary amination producing cationic KGM (CKGM), exhibited good biocompatibility, thermal stability, antibacterial activity, and pH-dependent drug release, with CKGM/SA/HA@DOX showing slightly lower cumulative release than KGM/SA/HA@DOX [125]. Additionally, alginate–KGM core–shell aerogel particles fabricated via air-assisted coaxial prilling and optimized with artificial neural networks and genetic algorithms displayed high surface area (201 ± 10 m^2^/g), macroporous–mesoporous structures, and efficient drug loading with vancomycin or dexamethasone, though burst release profiles indicated a need for coating modifications to achieve controlled release [126].

LBG, a natural galactomannan polysaccharide, is widely applied in food, pharmaceutical, and biomedical fields due to its flexibility, hydrogen-bonding capacity, and film-forming properties [127]. In food packaging, LBG-based films synergize with other biopolymers and bioactive agents to enhance antibacterial, antioxidant, and barrier properties, extending shelf life and freshness monitoring [125]. For biomedical applications, LBG composites show strong potential in drug delivery and wound healing owing to their mucoadhesive, swelling, and gel-forming properties. Modifications such as crosslinking and carboxymethylation further support controlled, targeted, and responsive delivery [128]. A nanocomposite hydrogel of LBG, poly(4-acryloylmorpholine), and silver nanoparticles demonstrated pH-responsive 5-fluorouracil release, antibacterial activity, and biocompatibility, highlighting promise for cancer therapy and infection control [129]. Additionally, LBG microparticles (~4 μm) have shown physicochemical stability across suppliers, >80% viability in respiratory cells, and a safe in vivo inhalation profile, supporting its development as a novel excipient for lung drug delivery [130].

Exopolysaccharide fractions from the hadal bacterium *Psychrobacter pulmonis*, particularly those rich in mannose (XL-1-D and XMRS-1-D), exhibited strong bioactivities. Mannose-containing fractions enhanced macrophage proliferation, phagocytosis, NO and reactive oxygen species production, and proinflammatory cytokine secretion, while also inhibiting A549 cancer cell proliferation via apoptosis-related pathways. These findings highlight mannose-rich microbial polysaccharides as promising candidates for drug development [131]. In conclusion, carbohydrate polymers such as mannans, guar gum, KGM, and LBG show great promise in drug delivery, wound healing, tissue engineering, and cancer therapy owing to their biocompatibility, biodegradability, and tunable properties. Advances in modification, nanotechnology, and stimuli-responsive systems enable targeted, sustained, and colon-specific release, improving therapeutic efficacy while reducing toxicity. Their versatility across oral, transdermal, pulmonary, and injectable platforms, along with emerging bioactivities like immunomodulation and anticancer effects, highlights their potential as next-generation biomaterials for precision medicine.

### 6.3. Biomedical and Tissue Engineering Applications

**Figure 4:**
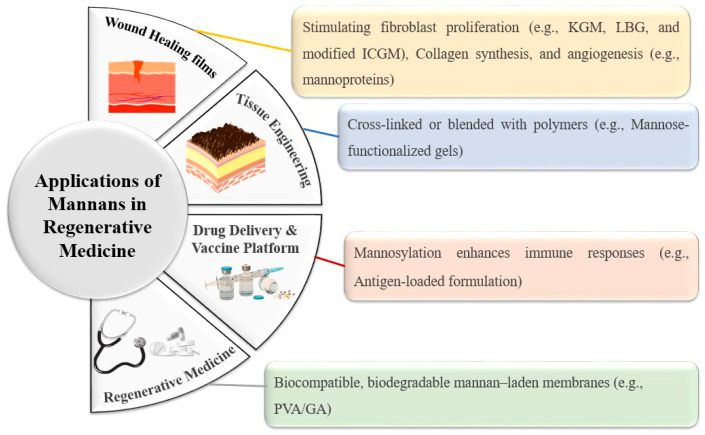
The various portals of mannan applications in tissue engineering. LBG: Locust Bean Gum; KGM: Konjac Glucomannan; ICGM: Ionically Crosslinked Galactomannan; PVA/GA: Polyvinyl Alcohol/Galactomannan.

Mannans are promising biomaterials for regenerative medicine, with applications in wound dressings, hydrogels, and scaffolds due to their ability to stimulate fibroblast proliferation, collagen synthesis, and angiogenesis, thereby accelerating wound healing [98]. In tissue engineering, mannans can be crosslinked or blended with polymers to create scaffolds that support cell growth and differentiation [9], while their immunomodulatory properties enhance potential in cancer therapy and vaccine adjuvant development [6,132]. Guar gum, derived from *Cyamopsis tetragonoloba* seeds, is low-cost, biocompatible, biodegradable, and widely available, but its uncontrolled hydration and microbial susceptibility necessitate modifications to improve solubility, swelling, pH sensitivity, and antimicrobial/antioxidative activities, expanding its role in tissue engineering and regenerative medicine [9]. Mannosylation has also been applied in vaccine platforms: HIV p24 antigen-loaded proteoliposomes functionalized with mannans enhanced dendritic cell uptake, MHCI/MHCII presentation, and costimulatory marker expression, inducing both humoral and cytotoxic T-cell responses [133]. Clinically, acemannan from Aloe vera improved short-term buccal bone stability in guided bone regeneration with simultaneous implant placement, showing safety but requiring further evaluation of long-term benefits [134]. Novel KGM-based hydrogels have also advanced wound healing: a KGM–*Bletilla striata* polysaccharide hydrogel crosslinked with 4-Butanediol Diglycidyl Ether showed superior water retention, mechanical strength, anti-inflammatory effects (via TNF-α/NF-κB inhibition and IL-10 upregulation), and enhanced angiogenesis and collagen deposition in vivo [135]. Similarly, a lipoic acid-modified KGM adhesive hydrogel delivering small interfering RNA of *alpha* cardiac muscle 1 effectively reduced fibroblast proliferation and collagen deposition, inhibiting keloid growth in mice [136]. Furthermore, polyvinyl alcohol/galactomannan asymmetric membranes crosslinked with sulfosuccinic acid demonstrated antibacterial, biodegradable, and biocompatible properties, with 20% galactomannan content enhancing fibroblast/osteoblast proliferation and reducing monocyte chemoattractant protein-1secretion, highlighting their potential in wound healing and tissue engineering [137]. In conclusion, mannans and their derivatives are versatile, biocompatible biomaterials with applications in wound healing, tissue engineering, drug delivery, and vaccine development. Their modifiable properties such as controlled swelling, antimicrobial activity, and immunomodulation support targeted and regenerative therapies, highlighting their strong potential for next-generation biomedical applications (Figure 4).

### 6.4. Environmental and Industrial Biotechnology

Mannans and their derivatives play diverse roles across environmental and industrial sectors. In environmental applications, their hydroxyl and carboxyl groups enable efficient binding of heavy metals and dyes, making them valuable in bioremediation, wastewater treatment, and biosorption [22]. Guar gum and xanthan gum nanocomposites provide eco-friendly, low-cost, and reusable adsorbents with high efficiency in pollutant removal, functioning through adsorption and degradation [138]. Guar gum itself has been modified into multifunctional adsorbents and nanocomposites capable of removing dyes, metals, oil, and pathogens, with recent advances demonstrating significant improvements in water quality [139]. In packaging and food preservation, KGM has emerged as a non-toxic, biodegradable, and film-forming biopolymer. KGM-based films enhance food shelf-life by improving moisture retention, gas barrier properties, and antimicrobial activity. Despite challenges such as limited mechanical strength and humidity sensitivity, blending with other biopolymers or adding nanoparticles improves performance, making KGM films a promising, eco-friendly alternative to plastics [140]. Modified mannans, more broadly are also explored as biodegradable packaging materials with the potential to replace petroleum-based plastics [12]. In biomedical and pharmaceutical applications, galactomannans (guar, locust bean, fenugreek gums) are widely studied for drug delivery and as pharmaceutical excipients. Developed into tablets, microparticles, nanoparticles, micelles, and hydrogels, these systems support oral vaccination, insulin delivery, cancer targeting, wound healing, and controlled release. Additionally, galactomannans act as green reducing agents for the synthesis of metal nanoparticle with enhanced antimicrobial, antioxidant, and anticancer properties, though lesser-studied sources (cassia, tara, Delonix) remain underexplored [116]. Protein–polysaccharide nanoconjugates, including those with mannans and glucomannans, further expand biomedical and food nanotechnology, improving nutraceutical stability and delivery [35]. In agriculture and construction, guar gum has been chemically modified into GG-PAM, a soil-strengthening biopolymer with improved thermal stability, mechanical strength, and erosion resistance. GG-PAM–treated soils demonstrated superior compressive strength, crack prevention, and durability under harsh conditions, supporting its use in roadbed construction and open-air infrastructure [125]. In biosensing, mannans enable innovative food safety tools. A biosensor based on mannose-functionalized AuNPs–mannan oligosaccharide nanozyme combined with split aptamers achieved highly sensitive detection of His-tagged proteins (limit: 12.44 nM), highlighting potential applications in monitoring synthetic biomolecules in food systems [141]. In summary, mannans function as versatile biopolymers bridging natural bioactivity with industrial utility. Their multifunctionality spans wastewater purification, eco-friendly packaging, drug delivery, agricultural reinforcement, food nanotechnology, and biosensing.

### 6.5. Safety of Mannans

Growing populations and resource concerns have driven the search for alternative food ingredients. Wood-derived hemicelluloses, such as softwood GGM, are abundant and exhibit high potential as multifunctional food additives, for example, as emulsion stabilizers in processed foods. Before market introduction, their safety must be evaluated under Novel Food regulations [142]. In this context, mannan-derived products, including β-mannanase and related enzymes, generally demonstrate a trustful safety profile across toxicological evaluations. For instance, a 90-day repeated-dose oral toxicity study conducted by the European Food Safety Authority (EFSA) on mannan endo-1,4-β-mannosidase derived from *Aspergillus niger* NZYM-NM proved a no-observed-adverse-effect level (NOAEL) of 100 mg total organic solids (TOS)/kg body weight/day, corresponding to a margin of exposure greater than 1100 [143]. Similarly, EFSA’s evaluation of mannanase derived from *A. niger* strain AE-HCM reported a NOAEL of 728 mg TOS/kg bw/day, again indicating a high safety margin relative to estimated dietary exposure [144]. Additional assessments of mannanase enzyme preparations for feed applications also consistently conclude that these products pose no consumer or animal health risks when used under recommended conditions [145].

Although systemic toxicity is low, the potential for allergenicity or respiratory sensitization requires attention, particularly for enzyme preparations. EFSA evaluation of the AE-HCM strain identified sequence similarity between mannanase and known respiratory allergens, suggesting that allergic sensitization cannot be completely excluded despite its low probability in typical dietary uses [144]. Feed additive assessments report similar concerns: mannanase-based products such as Natupulse^®^ TS and Hemicell^®^-L are not classified as irritants but may act as respiratory or skin sensitizers due to their proteinaceous nature [145,146]. These observations emphasize the importance of monitoring occupational exposure and immunogenic potential in industrial and feed settings that employ mannanases for producing mannan products. More interestingly, allergoid-mannan conjugates are a novel class of vaccines prescribed for allergen-specific immunotherapy, currently being evaluated in phase 2 clinical trials. These conjugates target dendritic cells (DCs), generating functional FOXP3-positive regulatory T (Treg) cells, and have been shown to reprogram monocyte differentiation into stable tolerogenic DCs through epigenetic and metabolic mechanisms, shedding light on their potential to induce allergen tolerance [147]. Polymerized allergoids conjugated with mannan have been developed specifically for grass pollen (*Phleum pratense* and *Dactylis glomerata*), and dose-finding studies indicate that both subcutaneous and sublingual administration at 3000–5000 mTU/mL is safe and efficacious in achieving the primary immunotherapeutic outcomes [148]. This approach represents a promising strategy for targeted modulation of the immune response in allergen immunotherapy using mannans.

The safe and effective dose range of mannans and mannan-derived materials varies widely depending on their intended application. For food enzyme use, EFSA estimates human exposure to mannanase preparations to be below 1 mg TOS/kg bw/day, far below toxicological thresholds determined in animal studies [149]. Non-European regulatory bodies also support the safety of mannan-based products. For example, the U.S. Food and Drug Administration affirmed the generally recognized as safe (GRAS) status of a β-mannanase preparation from *Aspergillus niger* for use in food processing, indicating acceptable safety under intended conditions of use [150]. Collectively, existing toxicological and regulatory evidence confirms that mannans and their derivatives are safe across a broad dose range, though novel biomedical or therapeutic applications will require dedicated long-term toxicity, immunogenicity, and clinical validation studies.

## 7. Future Prospects and Challenges of Mannans

Mannans are increasingly recognized as multifunctional biopolymers with applications spanning food, feed, packaging biomaterials, and biomedical fields. Despite their potential, their commercial translation remains constrained by scientific, industrial, and regulatory hurdles. Recent market analyses forecast steady growth, with the global MOS market projected to expand at a compound annual growth rate of 4.8–8.5% between 2025 and 2031, primarily driven by their use as feed additives and gut-health promoters in livestock and companion animals [151,152]. Key opportunities lie in expanding applications for animal feed, nutraceuticals, and functional foods, supported by regulatory restrictions on antibiotic growth promoters and increasing consumer demand for natural prebiotics. Advances in enzyme technology and green extraction methods also promise to improve sustainability and cost efficiency. Furthermore, diversifying raw material sources, such as forestry residues, microbial biomass, and agricultural byproducts, could reduce reliance on traditional feedstocks and stabilize supply chains. Nevertheless, these positive trends, significant challenges remain. Raw material volatility and dependence on crops such as guar, locust bean, and konjac expose the market to climatic, environmental, and geopolitical risks [153]. In addition, mannans must compete with more established prebiotics such as fructooligosaccharides and inulin, necessitating improved cost competitiveness and stronger evidence of superior health outcomes [154]. Regulatory heterogeneity across regions and limited clinical validation continue to hinder broader adoption in food and pharmaceutical applications.

Beyond these aforementioned barriers, a range of fundamental scientific questions continues to limit progress. Structure–function relationships in mannans, particularly how fine-scale branching patterns, acetylation levels, and molecular weight distributions influence biological activity, remain poorly resolved. This information gap constrains bioengineered design of mannans for targeted outcomes. Similarly, the biosynthesis, transport, and remodeling of mannans in plants, fungi, and microbes are not yet fully investigated, complicating endeavors to engineer organisms for predictable, high-yield production. Moreover, in non-food sectors, mannans’ inherent limitations in mechanical strength, thermal stability, and water resistance restrict their direct use in packaging and biomedical devices, requiring chemical modification, blending, or incorporation of nanomaterials to achieve desired performance [101]. Future progress will require a coordinated multidisciplinary approach. Synthetic biology and enzyme engineering could enable biosynthesis of mannans with tailored structural features and consistent functionality. Nanotechnology and advanced delivery systems may unlock biomedical potential through targeted, responsive, and safe formulations. Parallel efforts in green processing and supply-chain development are critical to ensuring scalability and economic feasibility. Finally, consumer education and clearer regulatory frameworks will be necessary to translate laboratory advances into market adoption. In summary, while mannans are strategically positioned as next-generation sustainable biomaterials, realizing their full industrial and clinical potential will depend on addressing challenges of standardization, cost competitiveness, and functional optimization. With continued innovation at the interface of plant biotechnology, materials science, and biomedical engineering, mannans could emerge as a cornerstone of the bioeconomy.

## 8. Conclusions and Remarks

Mannans are versatile, bioactive biopolymers widely distributed in plants, microbes, and marine organisms, exhibiting diverse structural features and physicochemical properties that strengthen their broad biological functions, including prebiotic, immunomodulatory, antimicrobial, antioxidant, anticancer, and tissue-regenerative activities. Their derivatives, such as galactomannans, glucomannans, and mannan-oligosaccharides, have found applications across food, feed, nutraceutical, pharmaceutical, and biomedical industries, as well as in environmental and industrial biotechnology. Despite their potential, challenges such as raw material variability, structural heterogeneity, limited mechanical and thermal properties, and gaps in clinical validation restrict large-scale utilization. Future research integrating enzyme engineering, green extraction methods, nanotechnology, and synthetic biology could enable the production of mannans with tailored structures and enhanced functionalities, expanding their applications in sustainable biomaterials, functional foods, therapeutics, and industrial processes. Overall, mannans represent a promising class of multifunctional biopolymers with strong potential to contribute to the bioeconomy, that is, economic systems that utilize renewable biological resources to produce materials, chemicals, and health products. Examples include bio-based packaging, biodegradable medical materials, microbial fermentation platforms, and natural prebiotic ingredients for food and feed. Through continued innovation, mannans could help advance renewable, low-carbon, and biologically derived product value chains.

## Figures and Tables

**Table 1:** Structure and structural properties of natural and canonicalized mannans were curated from https://pubchem.ncbi.nlm.nih.gov/, accessed on 12 June 2025.

	Name/PubChem CID	Molecular Weight g/mol	XLogP3-AA	Hydrogen Bond Donor Count	Hydrogen Bond Acceptor Count	Rotatable Bond Count	Topological Polar Surface Area Å
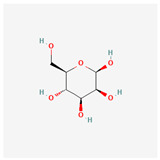 C_6_H_12_O_6_	*beta*-D-mannopyranose, *beta*-D-Mannose/439680	180.16	−2.6	5	6	1	110
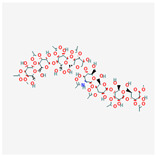 C_66_H_101_NO_49_	Acemannan, Cello gel, Acemannan (Aloe vera)/134129847	1692.5	−12.8	17	49	39	711
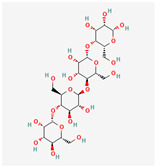 C_24_H_42_O_21_	*alpha*-D-Mannan, Mannan, Mannoglycan/25147451	666.6	−9	14	21	10	348
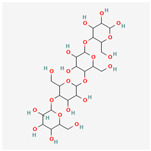 C_24_H_42_O_21_	Amylotetraose; Fujioligo 450; *alpha*-1,4-Tetraglucose/870	666.6	−9	14	21	10	348
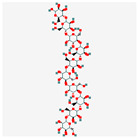 C_60_H_102_O_51_	1,4-b-D-Mannan, GlyTouCan: G45304DG, G45304DG/53477899	1639.4	−21.9	32	51	28	823
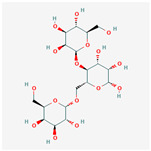 C_18_H_32_O_16_	D-Galacto-d-mannan/439336	504.4	−6.3	11	16	7	269
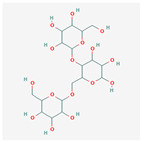 C_18_H_32_O_16_	GlyTouCan: G55283BR, Carob Galactomannan, 6-*O*-Glucosylmaltose, *Aspergillus fumigatus* galactomannan/3514701	504.4	−6.3	11	16	7	269

## Data Availability

No new data were created or analyzed in this study. Data sharing is not applicable to this article.

## References

[1] Gong M., Bassi A. (2016). Carotenoids from microalgae: A review of recent developments. Biotechnol. Adv..

[2] Liepman A.H., Nairn C.J., Willats W.G., Sørensen I., Roberts A.W., Keegstra K. (2007). Functional genomic analysis supports conservation of function among cellulose synthase-like a gene family members and suggests diverse roles of mannans in plants. Plant Physiol..

[3] Moreira L.R., Filho E.X. (2008). An overview of mannan structure and mannan-degrading enzyme systems. Appl. Microbiol. Biotechnol..

[4] Srivastava P.K., Kapoor M. (2017). Production, properties, and applications of endo-beta-mannanases. Biotechnol. Adv..

[5] Zhang R., Li B., Zhao Y., Zhu Y., Li L. (2024). An essential role for mannan degradation in both cell growth and secondary cell wall formation. J. Exp. Bot..

[6] Hall R.A., Gow N.A. (2013). Mannosylation in *Candida albicans*: Role in cell wall function and immune recognition. Mol. Microbiol..

[7] Cottier F., Hall R.A. (2019). Face/Off: The Interchangeable Side of *Candida albicans*. Front. Cell. Infect. Microbiol..

[8] Patel S., Goyal A. (2015). Applications of natural polymer gum Arabic: A review. Int. J. Food Prop..

[9] Teli S., Deshmukh K., Khan T., Suvarna V. (2024). Recent Advances in Biomedical Applications of Mannans and Xylans. Curr. Drug Targets.

[10] Voiniciuc C. (2022). Modern mannan: A hemicellulose’s journey. New Phytol..

[11] Hu Q., Huang G., Huang H. (2025). Extraction, structure, activity and application of konjac glucomannan. Ultrason. Sonochem..

[12] Scheller H.V., Ulvskov P. (2010). Hemicelluloses. Annu. Rev. Plant Biol..

[13] Cerqueira M., Bourbon A., Pinheiro A., Martins J., Souza B., Teixeira J., Vicente A. (2011). Galactomannans use in the development of edible films/coatings for food applications. Trends Food Sci. Technol..

[14] Petitjean M., Isasi J.R. (2022). Locust Bean Gum, a Vegetable Hydrocolloid with Industrial and Biopharmaceutical Applications. Molecules.

[15] Li F., Zhao J., Wei Y., Jiao X., Li Q. (2021). Holistic review of polysaccharides isolated from pumpkin: Preparation methods, structures and bioactivities. Int. J. Biol. Macromol..

[16] Zhong R., Zhou D., Chen L., Rose J.P., Wang B.C., Ye Z.H. (2024). Plant Cell Wall Polysaccharide O-Acetyltransferases. Plants.

[17] Sánchez R.A.R., Matulewicz M.C., Ciancia M. (2022). NMR spectroscopy for structural elucidation of sulfated polysaccharides from red seaweeds. Int. J. Biol. Macromol..

[18] Casillo A., Fabozzi A., Russo Krauss I., Parrilli E., Biggs C.I., Gibson M.I., Lanzetta R., Appavou M.S., Radulescu A., Tutino M.L. (2021). Physicochemical Approach to Understanding the Structure, Conformation, and Activity of Mannan Polysaccharides. Biomacromolecules.

[19] Xia P., Zheng Y., Sun L., Chen W., Shang L., Li J., Hou T., Li B. (2024). Regulation of glycose and lipid metabolism and application based on the colloidal nutrition science properties of konjac glucomannan: A comprehensive review. Carbohydr. Polym..

[20] Buckeridge M.S. (2010). Seed cell wall storage polysaccharides: Models to understand cell wall biosynthesis and degradation. Plant Physiol..

[21] Stephen A., Aspinall G.O. (1983). Other plant polysaccharides. The Polysaccharides.

[22] Cunha L., Grenha A. (2016). Sulfated seaweed polysaccharides as multifunctional materials in drug delivery applications. Mar. Drugs.

[23] Reid J.S.G., Edwards M., Kigel J., Galili G. (1995). Galactomannans and Other Cell Wall Storage Polysaccharides in Seeds. Seed Development and Germination.

[24] Tedjani A., Mouane A., Azzi M., Boual Z., Atoki A.V., Messaoudi M. (2025). Galactomannans from Chemical Insights to Multifaceted Applications: A Review. Nat. Prod. Commun..

[25] Stephen A.M., Phillips G.O. (2016). Food Polysaccharides and Their Applications.

[26] Shao J., Pu J., Chen F., Liu Y., Song J. (2025). Konjac glucomannan-based hydrogels with tunable mechanical strength and frictional resistance for biomedical applications. Int. J. Biol. Macromol..

[27] Wang Y., Yang C., Zhang W., Wang X., Zhao Z., Wang Z., Zhang L. (2025). Multifunctional self-healing and pH-responsive hydrogel dressing based on cationic guar gum and hyaluronic acid for on-demand drug release. Int. J. Biol. Macromol..

[28] Nagano T., Higashimura Y., Nakano M., Nishiuchi T., Lelo A.P. (2025). High-viscosity dietary fibers modulate gut microbiota and liver metabolism to prevent obesity in high-fat diet-fed mice. Int. J. Biol. Macromol..

[29] Sittikijyothin W., Torres D., Gonçalves M. (2005). Modelling the rheological behaviour of galactomannan aqueous solutions. Carbohydr. Polym..

[30] Fu K., Wang H., Pan T., Cai Z., Yang Z., Liu D., Wang W. (2024). Gel-forming polysaccharides of traditional gel-like foods: Sources, structure, gelling mechanism, and advanced applications. Food Res. Int..

[31] Huynh N., Fliri L., Valle-Delgado J.J., Österberg M. (2025). Exploiting the high affinity between cellulose nanofibrils and Aloe vera acemannan to develop elastic, crosslinker-free, all-polysaccharide hydrogels. Int. J. Biol. Macromol..

[32] Dakia P.A., Blecker C., Robert C., Wathelet B., Paquot M. (2008). Composition and physicochemical properties of locust bean gum extracted from whole seeds by acid or water dehulling pre-treatment. Food Hydrocoll..

[33] Wang P., Pei X., Zhou W., Zhao Y., Gu P., Li Y., Gao J. (2024). Research and application progress of microbial beta-mannanases: A mini-review. World J. Microbiol. Biotechnol..

[34] Ferreira S.A., Oslakovic C., Cukalevski R., Frohm B., Dahlbäck B., Linse S., Gama F.M., Cedervall T. (2012). Biocompatibility of mannan nanogel—Safe interaction with plasma proteins. BBA Gen. Subj..

[35] Paliya B.S., Sharma V.K., Sharma M., Diwan D., Nguyen Q.D., Aminabhavi T.M., Rajauria G., Singh B.N., Gupta V.K. (2023). Protein-polysaccharide nanoconjugates: Potential tools for delivery of plant-derived nutraceuticals. Food Chem..

[36] Wu J., Liu Y., Liu R., Xiao C., Xuan L., Wu L., Qian J., Qin X., Hou Y., Xie M. (2025). Fishing out AIEC with FimH capturing microgels for inflammatory bowel disease treatment. Nat. Commun..

[37] Salehi F. (2020). Effect of common and new gums on the quality, physical, and textural properties of bakery products: A review. J. Texture Stud..

[38] Willför S., Sundberg K., Tenkanen M., Holmbom B. (2008). Spruce-derived mannans—A potential raw material for hydrocolloids and novel advanced natural materials. Carbohydr. Polym..

[39] Sang J., Zhao G., Koidis A., Wei X., Huang W., Guo Z., Wu S., Huang R., Lei H. (2024). Isolation, structural, biological activity and application of *Gleditsia* species seeds galactomannans. Carbohydr. Polym..

[40] Malgas S., van Dyk J.S., Pletschke B.I. (2015). A review of the enzymatic hydrolysis of mannans and synergistic interactions between β-mannanase, β-mannosidase and α-galactosidase. World J. Microbiol. Biotechnol..

[41] Yu W., Shen L., Qi J., Hu T. (2022). Conjugation with loxoribine and mannan improves the immunogenicity of *Mycobacterium tuberculosis* CFP10-TB10.4 fusion protein. Eur. J. Pharm. Biopharm..

[42] Wang Y., Liu J., Li Q., Wang Y., Wang C. (2015). Two natural glucomannan polymers, from Konjac and *Bletilla*, as bioactive materials for pharmaceutical applications. Biotechnol. Lett..

[43] Granato D., Reshamwala D., Korpinen R., Azevedo L., do Carmo M.A.V., Cruz T.M., Marques M.B., Wen M., Zhang L., Marjomäki V. (2022). From the forest to the plate—Hemicelluloses, galactoglucomannan, glucuronoxylan, and phenolic-rich extracts from unconventional sources as functional food ingredients. Food. Chem..

[44] Kilpatrick D.C. (2002). Mannan-binding lectin and its role in innate immunity. Transfus. Med..

[45] Schiavone M., François J.M., Zerbib D., Capp J.-P. (2023). Emerging relevance of cell wall components from non-conventional yeasts as functional ingredients for the food and feed industry. Curr. Res. Food Sci..

[46] Méndez-Líter J.A., de Eugenio L.I., Nieto-Domínguez M., Prieto A., Martínez M.J. (2021). Hemicellulases from Penicillium and Talaromyces for lignocellulosic biomass valorization: A review. Bioresour. Technol..

[47] Wang Z.A., Li L.X., Doering T.L. (2018). Unraveling synthesis of the cryptococcal cell wall and capsule. Glycobiology.

[48] Zou Y., Liu M., Lai Y., Liu X., Li X., Li Y., Tang Q., Xu W. (2023). The glycoside hydrolase gene family profile and microbial function of *Debaryomyces hansenii* Y4 during South-road dark tea fermentation. Front. Microbiol..

[49] Liu Q., Xi Y., Wang Q., Liu J., Li P., Meng X., Liu K., Chen W., Liu X., Liu Z. (2021). Mannan oligosaccharide attenuates cognitive and behavioral disorders in the 5xFAD Alzheimer’s disease mouse model via regulating the gut microbiota-brain axis. Brain Behav. Immun..

[50] Shibata N., Suzuki A., Kobayashi H., Okawa Y. (2007). Chemical structure of the cell-wall mannan of *Candida albicans* serotype A and its difference in yeast and hyphal forms. Biochem. J..

[51] Larsbrink J., Tuveng T.R., Pope P.B., Bulone V., Eijsink V.G., Brumer H., McKee L.S. (2017). Proteomic insights into mannan degradation and protein secretion by the forest floor bacterium *Chitinophaga pinensis*. J. Proteom..

[52] Synytsya A., Čopíková J., Kim W.J., Park Y.I. (2015). Cell wall polysaccharides of marine algae. Springer Handbook of Marine Biotechnology.

[53] Grice I.D., Mariottini G.L. (2018). Glycans with Antiviral Activity from Marine Organisms. Marine Organisms as Model Systems in Biology and Medicine.

[54] Wang L., Wang X., Wu H., Liu R. (2014). Overview on biological activities and molecular characteristics of sulfated polysaccharides from marine green algae in recent years. Mar. Drugs.

[55] Chi Y., Li Y., Ding C., Liu X., Luo M., Wang Z., Bi Y., Luo S. (2024). Structural and biofunctional diversity of sulfated polysaccharides from the genus *Codium* (Bryopsidales, Chlorophyta): A review. Int. J. Biol. Macromol..

[56] Chauhan P.S., Gupta N. (2017). Insight into microbial mannosidases: A review. Crit. Rev. Biotechnol..

[57] Dhawan S., Kaur J. (2007). Microbial mannanases: An overview of production and applications. Crit. Rev. Biotechnol..

[58] Chauhan P.S., Puri N., Sharma P., Gupta N. (2012). Mannanases: Microbial sources, production, properties and potential biotechnological applications. Appl. Microbiol. Biotechnol..

[59] Galante Y.M., Merlini L., Silvetti T., Campia P., Rossi B., Viani F., Brasca M. (2018). Enzyme oxidation of plant galactomannans yielding biomaterials with novel properties and applications, including as delivery systems. Appl. Microbiol. Biotechnol..

[60] Faustino M., Durão J., Pereira C.F., Pintado M.E., Carvalho A.P. (2021). Mannans and mannan oligosaccharides (MOS) from *Saccharomyces cerevisiae*–A sustainable source of functional ingredients. Carbohydr. Polym..

[61] Rana M., Jassal S., Yadav R., Sharma A., Puri N., Mazumder K., Gupta N. (2024). Functional β-mannooligosaccharides: Sources, enzymatic production and application as prebiotics. Crit. Rev. Food Sci. Nutr..

[62] Shoaf K., Mulvey G.L., Armstrong G.D., Hutkins R.W. (2006). Prebiotic galactooligosaccharides reduce adherence of enteropathogenic *Escherichia coli* to tissue culture cells. Infect. Immun..

[63] Utama G.L., Oktaviani L., Balia R.L., Rialita T. (2023). Potential application of yeast cell wall biopolymers as probiotic encapsulants. Polymers.

[64] Al-Asmari F., Abdelshafy A.M., Neetoo H., Zhang Y. (2025). Natural gums (gum Arabic, guar gum and xanthan gum) as a promising source of prebiotics: A review on their functional roles and food applications. Int. J. Biol. Macromol..

[65] Jana U.K., Suryawanshi R.K., Prajapati B.P., Kango N. (2021). Prebiotic mannooligosaccharides: Synthesis, characterization and bioactive properties. Food Chem..

[66] Kapoor D.U., Sharma H., Maheshwari R., Pareek A., Gaur M., Prajapati B.G., Castro G.R., Thanawuth K., Suttiruengwong S., Sriamornsak P. (2024). Konjac glucomannan: A comprehensive review of its extraction, health benefits, and pharmaceutical applications. Carbohydr. Polym..

[67] Tang N., Wang X., Yang R., Liu Z., Liu Y., Tian J., Xiao L., Li W. (2022). Extraction, isolation, structural characterization and prebiotic activity of cell wall polysaccharide from *Kluyveromyces marxianus*. Carbohydr. Polym..

[68] Wang J., Ke S., Strappe P., Ning M., Zhou Z. (2023). Structurally Orientated Rheological and Gut Microbiota Fermentation Property of Mannans Polysaccharides and Oligosaccharides. Foods.

[69] Kwak S., Robinson S.J., Lee J.W., Lim H., Wallace C.L., Jin Y.-S. (2022). Dissection and enhancement of prebiotic properties of yeast cell wall oligosaccharides through metabolic engineering. Biomaterials.

[70] Fernandes A., Nair A., Kulkarni N., Todewale N., Jobby R. (2023). Exploring Mushroom Polysaccharides for the Development of Novel Prebiotics: A Review. Int. J. Med. Mushrooms.

[71] Panwar D., Shubhashini A., Kapoor M. (2023). Complex alpha and beta mannan foraging by the human gut bacteria. Biotechnol. Adv..

[72] Dev K., Akbar Mir N., Biswas A., Kannoujia J., Begum J., Kant R. (2020). Dietary Mannan-oligosaccharides potentiate the beneficial effects of *Bifidobacterium bifidum* in broiler chicken. Lett. Appl. Microbiol..

[73] Corrigan A., McCooey P., Taylor-Pickard J., Stockdale S., Murphy R. (2024). Breaking the Cycle: A Yeast Mannan-Rich Fraction Beneficially Modulates Egg Quality and the Antimicrobial Resistome Associated with Layer Hen Caecal Microbiomes under Commercial Conditions. Microorganisms.

[74] Zhang Q., Li L., Ma Z., He W., Huang E., Meng L., Li L., Tong T., Yang H., Liu Y. (2025). Effects of Mannan Oligosaccharides on Growth, Antioxidant and Immune Performance, and mTOR Signaling Pathway in Juvenile Tilapia (*Oreochromis niloticus*). Animals.

[75] Brown G.D., Gordon S. (2005). Immune recognition of fungal beta-glucans. Cell. Microbiol..

[76] Almeida P., Alves I., Fernandes Â., Lima C., Freitas R., Braga I., Correia J., Jerónimo C., Pinho S.S. (2025). Mannose glycans as key players in trained immunity: A novel anti-tumoral catalyst. BBA Gen. Subj..

[77] Seibold F., Stich O., Hufnagl R., Kamil S., Scheurlen M. (2001). Anti-*Saccharomyces cerevisiae* antibodies in inflammatory bowel disease: A family study. Scand. J. Gastroenterol..

[78] Ma Z., Ensley H.E., Lowman D.W., Kruppa M.D., Williams D.L. (2025). Recent advances in chemical synthesis of phosphodiester linkages found in fungal mannans. Carbohydr. Res..

[79] Pan X., Zong Q., Liu C., Wu H., Fu B., Wang Y., Sun W., Zhai Y. (2024). Konjac glucomannan exerts regulatory effects on macrophages and its applications in biomedical engineering. Carbohydr. Polym..

[80] Song X., Lei T., Cui N., Jin X., Huang Y., Shi Y., Zhao Z. (2025). A preliminary investigation on the protective effects of beta-glucan and mannan induced trained immunity in pufferfish *Takifugu obscurus*. Fish Shellfish Immunol..

[81] Witvrouw M., De Clercq E. (1997). Sulfated polysaccharides extracted from sea algae as potential antiviral drugs. Gen. Pharmacol..

[82] Wang W., Wang S.X., Guan H.S. (2012). The antiviral activities and mechanisms of marine polysaccharides: An overview. Mar. Drugs.

[83] Mardani R., Bahmanje A., Kazeroni Y.C., Khoshroo F., Roshanaie B., Sadeghche T., Pajaie K., Hosseini S.N., Doroud D., Shahali M. (2025). Oxidized Mannan: A Novel Adjuvant Candidate for Enhancing Immune Responses in Veterinary Rabies Vaccine. Chonnam Med. J..

[84] Kim H.W., Ko M.-K., Park S.H., Shin S., Kim G.S., Kwak D.Y., Park J.-H., Kim S.-M., Lee J.-S., Lee M.J. (2024). D-galacto-D-mannan-mediated Dectin-2 activation orchestrates potent cellular and humoral immunity as a viral vaccine adjuvant. Front. Immunol..

[85] Pignard C., Schiller H., Seyffer A., Schülke S. (2024). Mannan-, VLP-, and flagellin-based adjuvants for allergen-specific immunotherapy: A review of the current literature. Allergo J. Int..

[86] Van Emmerik L., Kuijper E., Fijen C., Dankert J., Thiel S. (1994). Binding of mannan-binding protein to various bacterial pathogens of meningitis. Clin. Exp. Immunol..

[87] Ferket P., Parks C., Grimes J. Benefits of dietary antibiotic and mannanoligosaccharide supplementation for poultry. Proceedings of the Multi-State Poultry Meeting.

[88] Van Die I., Cummings R.D. (2017). The Mannose Receptor in Regulation of Helminth-Mediated Host Immunity. Front. Immunol..

[89] Leclercq E., Pontefract N., Rawling M., Valdenegro V., Aasum E., Andujar L.V., Migaud H., Castex M., Merrifield D. (2020). Dietary supplementation with a specific mannan-rich yeast parietal fraction enhances the gut and skin mucosal barriers of Atlantic salmon (*Salmo salar*) and reduces its susceptibility to sea lice (*Lepeophtheirus salmonis*). Aquaculture.

[90] Bonde C.S., Drohse F.B., Budeyri Gokgoz N., Krych L., Nielsen D.S., Petersen H.H., Matthiesen R., Pedersen N.R., Geldhof P., Williams A.R. (2025). Dietary supplementation with fermented rapeseed and seaweed modulates parasite infections and gut microbiota in outdoor pigs. Front. Vet. Sci..

[91] Prasanphanich N.S., Mickum M.L., Heimburg-Molinaro J., Cummings R.D. (2013). Glycoconjugates in host-helminth interactions. Front. Immunol..

[92] Im S.-A., Oh S.-T., Song S., Kim M.-R., Kim D.-S., Woo S.-S., Jo T.H., Park Y.I., Lee C.-K. (2005). Identification of optimal molecular size of modified *Aloe* polysaccharides with maximum immunomodulatory activity. Int. Immunopharmacol..

[93] Liu C., Cui Y., Pi F., Cheng Y., Guo Y., Qian H. (2019). Extraction, purification, structural characteristics, biological activities and pharmacological applications of acemannan, a polysaccharide from aloe vera: A review. Molecules.

[94] Qiang W. (2025). Yeast Mannan: A Comprehensive Functional Analysis from Structural Characteristics to Biological Activity. Carbohydr. Res..

[95] Fernández P.V., Estevez J.M., Cerezo A.S., Ciancia M. (2012). Sulfated β-d-mannan from green seaweed *Codium vermilara*. Carbohydr. Polym..

[96] Yao X., Wan X., Chi Y., Nie X. (2025). Preparation, structure, and biological activity of sulfated polysaccharides from green algae in the Cladophoraceae (Cladophorales, Ulvophyceae). Antonie Van Leeuwenhoek.

[97] Shih P.C., Lin C.H., Chokkalingam U., Prakash E., Kao C.N., Chang C.F., Lin W.L. (2024). The *Aloe vera* acemannan polysaccharides inhibit phthalate-induced cell viability, metastasis, and stemness in colorectal cancer cells. Ecotoxicol. Environ. Saf..

[98] Choi S., Chung M.-H. (2003). A review on the relationship between Aloe vera components and their biologic effects. Semin. Integr. Med..

[99] Negi D., Bhavya K., Pal D., Singh Y. (2024). Acemannan coated, cobalt-doped biphasic calcium phosphate nanoparticles for immunomodulation regulated bone regeneration. Biomater. Sci..

[100] Kaur S., Santra S. (2022). Application of Guar Gum and its Derivatives as Green Binder/Separator for Advanced Lithium-Ion Batteries. ChemistryOpen.

[101] Singh S., Singh G., Arya S.K. (2018). Mannans: An overview of properties and application in food products. Int. J. Biol. Macromol..

[102] Shen L., Haufe J., Patel M.K. (2009). Product Overview and Market Projection of Emerging Bio-Based Plastics.

[103] Ye S., Zongo A.W.S., Shah B.R., Li J., Li B. (2021). Konjac glucomannan (KGM), deacetylated KGM (Da-KGM), and degraded KGM derivatives: A special focus on colloidal nutrition. J. Agric. Food Chem..

[104] Abik F., Palasingh C., Bhattarai M., Leivers S., Strom A., Westereng B., Mikkonen K.S., Nypelo T. (2023). Potential of wood hemicelluloses and their derivates as food ingredients. J. Agric. Food Chem..

[105] Yasukawa Z., Inoue R., Ozeki M., Okubo T., Takagi T., Honda A., Naito Y. (2019). Effect of Repeated Consumption of Partially Hydrolyzed Guar Gum on Fecal Characteristics and Gut Microbiota: A Randomized, Double-Blind, Placebo-Controlled, and Parallel-Group Clinical Trial. Nutrients.

[106] Beteri B., Barone M., Turroni S., Brigidi P., Tzortzis G., Vulevic J., Sekulic K., Motei D.E., Costabile A. (2024). Impact of Combined Prebiotic Galacto-Oligosaccharides and *Bifidobacterium breve*-Derived Postbiotic on Gut Microbiota and HbA1c in Prediabetic Adults: A Double-Blind, Randomized, Placebo-Controlled Study. Nutrients.

[107] Biswas A., Mohan N., Dev K., Mir N.A., Tiwari A.K. (2021). Effect of dietary mannan oligosaccharides and fructo-oligosaccharides on physico-chemical indices, antioxidant and oxidative stability of broiler chicken meat. Sci. Rep..

[108] Kiarie E.G., Steelman S., Martinez M., Livingston K. (2021). Significance of single beta-mannanase supplementation on performance and energy utilization in broiler chickens, laying hens, turkeys, sows, and nursery-finish pigs: A meta-analysis and systematic review. Transl. Anim. Sci..

[109] Zha A., Tan B., Wang J., Qi M., Deng Y., Liao P., Yin Y. (2023). The nanocomposites of modified attapulgite with vitamin E and mannan oligosaccharide regulated the intestinal epithelial barrier and improved intestinal microbiota composition to prevent diarrhea in weaned piglets. J. Sci. Food. Agric..

[110] Dar A.H., Singh S.K., Rahman J.U., Ahmad S.F. (2022). The effects of probiotic *Lactobacillus acidophilus* and/or prebiotic mannan oligosaccharides on growth performance, nutrient utilization, blood metabolites, faecal bacteria, and economics of crossbred calves. Iran. J. Vet. Res..

[111] Grossi S., Dell’Anno M., Rossi L., Compiani R., Sgoifo Rossi C.A. (2021). Supplementation of Live Yeast, Mannan Oligosaccharide, and Organic Selenium during the Adaptation Phase of Newly Arrived Beef Cattle: Effects on Health Status, Immune Functionality, and Growth Performance. Antibiotics.

[112] Abd El-Aziz A.H., Mota-Rojas D., Akinjute O.F., Abioja M.O. (2025). Prebiotic Oligosaccharides as Potential Growth Promoter in Rabbits: A Review. J. Anim. Physiol. Anim. Nutr..

[113] Lu Z.Y., Jiang W.D., Wu P., Liu Y., Kuang S.Y., Tang L., Yang J., Zhou X.Q., Feng L. (2020). Mannan oligosaccharides supplementation enhanced head-kidney and spleen immune function in on-growing grass carp (*Ctenopharyngodon idella*). Fish Shellfish Immunol..

[114] Ghyselinck J., Verstrepen L., Rakebrandt M., Marynissen S., Daminet S., Marzorati M. (2025). In vitro fermentation of yeast cell walls (mannan-oligosaccharide) and purified beta-glucans modulates the colonic microbiota of dogs with inflammatory bowel disease and demonstrates protective effects on barrier integrity and anti-inflammatory properties. PLoS ONE.

[115] Udaipuria N., Bhattacharya S. (2025). Novel Carbohydrate Polymer-Based Systems for Precise Drug Delivery in Colon Cancer: Improving Treatment Effectiveness With Intelligent Biodegradable Materials. Biopolymers.

[116] Yadav H., Maiti S. (2020). Research progress in galactomannan-based nanomaterials: Synthesis and application. Int. J. Biol. Macromol..

[117] Irfan J., Ali A., Hussain M.A., Haseeb M.T., Alsahli T.G., Naeem-Ul-Hassan M., Tulain U.R., Hussain S.Z., Hussain I., Azhar I. (2024). A superabsorbent and pH-responsive copolymer-hydrogel based on acemannan from *Aloe vera* (*Aloe barbadensis* M.): A smart material for drug delivery. Int. J. Biol. Macromol..

[118] Madrid R.R.M., Mathews P.D., Pramanik S., Mangiarotti A., Fernandes R., Itri R., Dimova R., Mertins O. (2024). Hybrid crystalline bioparticles with nanochannels encapsulating acemannan from *Aloe vera*: Structure and interaction with lipid membranes. J. Colloid Interface Sci..

[119] Verma D., Sharma S.K. (2021). Recent advances in guar gum based drug delivery systems and their administrative routes. Int. J. Biol. Macromol..

[120] Amjed N., Zeshan M., Farooq A., Naz S. (2024). Applications of guar gum polysaccharide for pharmaceutical drug delivery: A review. Int. J. Biol. Macromol..

[121] Lin J., Sun Y., Santos H.O., Gaman M.A., Bhat L.T., Cui Y. (2021). Effects of guar gum supplementation on the lipid profile: A systematic review and meta-analysis of randomized controlled trials. Nutr. Metab. Cardiovasc. Dis..

[122] Manna S., Karmakar S., Sen O., Sinha P., Jana S., Jana S. (2024). Recent updates on guar gum derivatives in colon specific drug delivery. Carbohydr. Polym..

[123] Zhuang K., Shu X., Xie W. (2024). Konjac glucomannan-based composite materials: Construction, biomedical applications, and prospects. Carbohydr. Polym..

[124] Zhao Z., Shi W., Wu Y., Kong L., Gao J., Kong Y. (2025). A stimuli-responsive drug delivery system based on konjac glucomannan, carboxymethyl chitosan and mesoporous polydopamine nanoparticles. Int. J. Biol. Macromol..

[125] Liu Y., Du C., Yi F., Li J., Li M. (2025). Enhancement on durability of cohesive soil by solidifying with modified guar gum. Int. J. Biol. Macromol..

[126] Illanes-Bordomás C., Landin M., García González C.A. (2025). Novel Core–Shell Aerogel Formulation for Drug Delivery Based on Alginate and Konjac Glucomannan: Rational Design Using Artificial Intelligence Tools. Polymers.

[127] Sana S.S., Raorane C.J., Venkatesan R., Roy S., Swain S.K., Kim S.C., Al-Tabakha M., Bhandare R.R., Raj V., Lee S. (2024). State-of-the-art progress on locust bean gum polysaccharide for sustainable food packaging and drug delivery applications: A review with prospectives. Int. J. Biol. Macromol..

[128] Kumar D., Malviya R., Sridhar S.B., Shareef J., Wadhwa T. (2025). Extraction, Physicochemical Properties, and Biomedical Applications of Locust Bean Gum: A Comprehensive Review. Mini Rev. Med. Chem..

[129] Luanda A., Mahadev M., Charyulu R.N., Badalamoole V. (2024). Locust bean gum-based silver nanocomposite hydrogel as a drug delivery system and an antibacterial agent. Int. J. Biol. Macromol..

[130] Pontes J.F., Guerreiro F., Silva J.P., Almeida M.P., Rosso A., da Costa A.M.R., Agusti G., Lollo G., Gaspar M.M., Grenha A. (2025). Locust bean gum (LBG)—A potential excipient for inhalation purposes: Excipient characterisation and in vitro and in vivo toxicological evaluation. Carbohydr. Polym..

[131] Qi M., Yan S., Cui Y., Huang Y., Liu Y., Wu W., Yu X., Wang P. (2025). Mannan-Containing Polymers from Hadal Bacterium *Psychrobacter pulmonis*: Preparation, Structural Analysis, Immunological Activity and Antitumor Effects. Mar. Drugs.

[132] Zhang X., Zhang Z., Xia N., Zhao Q. (2021). Carbohydrate-containing nanoparticles as vaccine adjuvants. Expert Rev. Vaccines.

[133] Zachová K., Bartheldyová E., Hubatka F., Křupka M., Odehnalová N., Knötigová P.T., Vaškovicová N., Sloupenská K., Hromádka R., Paulovičová E. (2024). The immunogenicity of p24 protein from HIV-1 virus is strongly supported and modulated by coupling with liposomes and mannan. Carbohydr. Polym..

[134] Deesricharoenkiat N., Jansisyanont P., Chuenchompoonut V., Mattheos N., Thunyakitpisal P. (2022). The effect of acemannan in implant placement with simultaneous guided bone regeneration in the aesthetic zone: A randomized controlled trial. Int. J. Oral Maxillofac. Surg..

[135] Hao Y., Wang J., Zhang H., Liu Q., Wang X., Wei Y., Liang Z., Hu Y., Huang D. (2025). Konjac glucomannan/*Bletilla striata* polysaccharide composite hydrogel: A promising anti-inflammatory dressing for accelerated wound healing. Carbohydr. Polym..

[136] Zhong Y., Zhang Y., Liu Y., Zeng K., Fan L., Wang Q., Zhang J. (2025). Adhesive hydrogel based on Konjac Glucomannan (KGM) loaded with siACTC1-exosomes for enhanced post-surgical keloid treatment. Int. J. Biol. Macromol..

[137] Varguez-Catzim P., Hernandez-Aburto M., Rodriguez-Canto W., Hunh-Ibarra M., Aguilar-Vega M., Claudio-Rizo J.A., Gonzalez-Diaz M.O. (2025). Tailoring membrane technology with galactomannan for enhanced biocompatibility and antibacterial action. Int. J. Biol. Macromol..

[138] Usman M., Taj M.B., Carabineiro S.A.C. (2023). Gum-based nanocomposites for the removal of metals and dyes from waste water. Environ. Sci. Pollut. Res. Int..

[139] Mandal S., Hwang S., Shi S.Q. (2023). Guar gum, a low-cost sustainable biopolymer, for wastewater treatment: A review. Int. J. Biol. Macromol..

[140] Shi S., Huang H., Duan L., Xie X., Zhang J., Tang J., Liu W., Tong C., Pang J., Wu C. (2025). Konjac glucomannan-based films and coatings for food packaging: Advances, applications, and future perspectives. Carbohydr. Polym..

[141] Wei K., Zhang L., Li N., Gao K., Li X., Li J., Wang S., Mao X. (2025). A colorimetric biosensor composed of split aptamers and mannan oligosaccharide nanozyme to monitor synthetic His-tagged food biomolecules. Food Chem..

[142] Pitkänen L., Heinonen M., Mikkonen K.S. (2018). Safety considerations of plant polysaccharides for food use: A case study on phenolic-rich softwood galactoglucomannan extract. Food. Funct..

[143] Panel E.C. (2022). Safety evaluation of the food enzyme mannan endo-1, 4-β-mannosidase from the genetically modified *Aspergillus niger* strain NZYM-NM. EFSA J..

[144] Zorn H., Barat Baviera J.M., Bolognesi C., Catania F., Gadermaier G., Greiner R., Mayo B., Mortensen A., Roos Y.H. (2025). Safety evaluation of the food enzyme mannan endo-1, 4-β-mannosidase from the non-genetically modified *Aspergillus niger* strain AE-HCM. EFSA J..

[145] Bampidis V., Azimonti G., Bastos M.D.L., Christensen H., Durjava M., Dusemund B., Kouba M., López-Alonso M., López Puente S. (2024). Safety of a feed additive consisting of endo 1, 4 β-d-mannanase produced by *Thermothelomyces thermophilus* DSM 33149 (Natupulse® TS/TS L) for chickens and turkeys for fattening, minor poultry species for fattening and ornamental birds (BASF SE). EFSA J..

[146] Bampidis V., Azimonti G., Bastos M.D.L., Christensen H., Dusemund B., Kouba M., Kos Durjava M., López-Alonso M., López Puente S., Marcon F. (2019). Safety and efficacy of Hemicell®-L (endo-1, 4-β-mannanase) as a feed additive for chickens for fattening or reared for laying, turkeys for fattening or reared for breeding and minor poultry species. EFSA J..

[147] Benito-Villalvilla C., Pérez-Diego M., Angelina A., Kisand K., Rebane A., Subiza J.L., Palomares O. (2022). Allergoid–mannan conjugates reprogram monocytes into tolerogenic dendritic cells via epigenetic and metabolic rewiring. J. Allergy Clin. Immunol..

[148] Ojeda P., Barjau M.C., Subiza J., Moreno A., Ojeda I., Solano E., Alonso A., Caballero R., Del Pozo S., Gómez-Perosanz M. (2024). Grass pollen allergoids conjugated with mannan for subcutaneous and sublingual immunotherapy: A dose-finding study. Front. Immunol..

[149] Lambré C., Barat Baviera J.M., Bolognesi C., Cocconcelli P.S., Crebelli R., Gott D.M., Grob K., Lampi E., Mengelers M. (2023). Food manufacturing processes and technical data used in the exposure assessment of food enzymes. EFSA J..

[150] FDA (2018). GRAS Notice No. 739: β-Mannanase Enzyme Preparation from Aspergillus niger.

[151] Research Markets (2024). Mannan Oligosaccharides Market Report 2025–2031: Growth Drivers and Regional Analysis.

[152] Precision Business Insights (2024). Global Mannan Oligosaccharides Market: Growth, Trends, and Forecast (2025–2031).

[153] Pmarket Research (2024). Mannan Oligosaccharides (MOS) Market Size, Share, and Trends Analysis, 2024–2031.

[154] Karimi I., Ghowsi M., Mohammed L.J., Haidari Z., Nazari K., Schiöth H.B. (2025). Inulin as a Biopolymer; Chemical Structure, Anticancer Effects, Nutraceutical Potential and Industrial Applications: A Comprehensive Review. Polymers.

